# A Metabolomic Approach to Target Compounds from the Asteraceae Family for Dual COX and LOX Inhibition

**DOI:** 10.3390/metabo5030404

**Published:** 2015-07-08

**Authors:** Daniela A. Chagas-Paula, Tong Zhang, Fernando B. Da Costa, RuAngelie Edrada-Ebel

**Affiliations:** 1 University of Strathclyde, the John Arbuthnott Building, 161 Cathedral Street, Glasgow G4 0RE, UK; E-Mail: tong.zhang.101@strath.ac.uk; 2 School of Pharmaceutical Sciences of Ribeirão Preto (FCFRP), University of São Paulo (USP), Department of Pharmaceutical Sciences, Av. Café s/n, Ribeirão Preto-SP 14040-903, Brazil; E-Mail: febcosta@fcfrp.usp.br

**Keywords:** metabolomics, Asteraceae, COX, LOX, HPLC-ESI-HRMS, O2PLS

## Abstract

The application of metabolomics in phytochemical analysis is an innovative strategy for targeting active compounds from a complex plant extract. Species of the Asteraceae family are well-known to exhibit potent anti-inflammatory (AI) activity. Dual inhibition of the enzymes COX-1 and 5-LOX is essential for the treatment of several inflammatory diseases, but there is not much investigation reported in the literature for natural products. In this study, 57 leaf extracts (EtOH-H_2_O 7:3, *v*/*v*) from different genera and species of the Asteraceae family were tested against COX-1 and 5-LOX while HPLC-ESI-HRMS analysis of the extracts indicated high diversity in their chemical compositions. Using O2PLS-DA (*R*^2^ > 0.92; VIP > 1 and positive Y-correlation values), dual inhibition potential of low-abundance metabolites was determined. The O2PLS-DA results exhibited good validation values (cross-validation = *Q*^2^ > 0.7 and external validation = *P*^2^ > 0.6) with 0% of false positive predictions. The metabolomic approach determined biomarkers for the required biological activity and detected active compounds in the extracts displaying unique mechanisms of action. In addition, the PCA data also gave insights on the chemotaxonomy of the family Asteraceae across its diverse range of genera and tribes.

## 1. Introduction

According to the latest reviews on drug discovery [1,2,3,4,5,6], natural products are still the most successful source of biologically-active lead compounds, even when compared with advanced strategies such as high-throughput screening of substances obtained through synthesis and combinatorial chemistry. However, the traditional strategy has some disadvantages that act as obstacles in the study of natural products, such as its complexity and inherent slowness [2,6]. Traditional strategies used in natural products research are not always able to detect the true active compounds masked by less active major substances in crude plant extracts, or the utilized analytical approach identifies none or just merely some active components [7,8]. There are some cases in which the crude extract is more active than the isolated pure compound, e.g., the extract of *Artemisia annua* has more potent antimalarial properties than its pure natural product, artemisinin [9,10,11].

Metabolomics is a comprehensive strategy which allows profiling of a complex mixture of numerous chemical components in a crude extract as well as targeting substances that can be correlated to a certain biological activity before commencing any time-consuming isolation procedure [8,12,13,14,15]. The metabolomic tool is not a reductionist method aiming to find one active compound against a known target receptor [8,15]. Instead, it uses thorough and accurate hyphenated analytical techniques in conjunction with suitable multivariate statistical analysis (MSA) tools that are able to simultaneously evaluate a huge number of metabolites and determine their correlations with certain biological properties [8,15,16,17,18,19]. Several analytical techniques have been applied in metabolomics studies [16,20,21,22,23]. In combination with metabolomics, this allows rapid dereplication, which is the identification of known compounds from reference spectral databases [17,22]. In preparation to a rigorous targeted isolation procedure of novel bioactive compounds, an efficient dereplication study can save time and effort to isolate well-studied active compounds or redundant inactive natural products. Independent of which analytical techniques were chosen, the usually huge metabolomic data obtained would require MSA to classify the samples into different groups and to facilitate their interpretation in terms of metabolite distribution under distinct variables [15,24]. Among the types of MSA, Principal Component Analysis (PCA) and Orthogonal-orthogonal Partial Least Square-Discriminant Analysis (O2PLS-DA) are commonly used for this purpose [15,24,25]. PCA is an unsupervised method that is used to get a sample overview and distribution to observe trends and/or outliers by performing variable reduction [24]. On the other hand, supervised methods, such as PLS and O2PLS-DA, are employed to find X variables (e.g., compounds in different extracts) correlating with determined Y variables (e.g., biological properties, geographical origin, chromatographic retention times, *etc.*). Thus, PLS and O2PLS are powerful statistical tools to determine such (bio)markers. The O2PLS-DA algorithm is a PLS modification, where systematic variation is removed from the input data set X that does not correlate to the response set Y, and therefore providing models with reduced complexity and more relevant results that are easier to interpret [26,27].

The aim of this study is to systematically detect and identify anti-inflammatory (AI) active compounds directly from their crude Asteraceae plant extracts. The chosen group of plants from the Asteraceae family have been earlier investigated and described for their AI property [28,29,30,31]. Several species from Asteraceae are well-known as AI plants [30,32], such as arnica (*Arnica montana* L.) [33], feverfew (*Tanacetum parthenium* (L.) Sch. Bip.) [34,35], pot marigold (*Calendula officinalis* L.) [36] and chicory (*Cichorium intybus* L.) [37]. Cyclooxygenase (COX) and lipoxygenase (LOX) pathways are of utmost importance in inflammatory processes, and therefore dual inhibitors of enzymes COX-1 and 5-LOX would be potential AI medicines with higher efficacy and fewer side effects than any currently available non-steroidal AI drug(NSAID) [29,32,38,39,40,41,42]. NSAIDs are among the most administered drugs worldwide; however, there are still some inflammatory diseases wanting efficient and safe treatment, such as rheumatoid arthritis, Alzheimer's disease and atherosclerosis [30,39].

Ethanolic leaf extracts(EtOH-H_2_O 7:3, *v*/*v*) from 57 Asteraceae plant species (Table S1) have been earlier systematically evaluated for dual inhibition of COX-1 and 5-LOX-5 [28,29,30,31] (Table 1), and these same extracts were tested for the current metabolomics profiling studies. These Asteraceae plant extracts cover a substantial metabolome diversity that could be explored through sensitive and high resolution analytical techniques together with MSA to target AI compounds with dual inhibition properties. These metabolites were found to be non-volatile compounds ranging from high to intermediate polarity [29], which consist mainly of flavonoids, chlorogenic acid congeners and sesquiterpene lactones [29,30,31].

Thus, for these metabolites, high-performance LC coupled to high-resolution MS (HPLC-HRMS), in reversed-phase chromatography and electrospray ionization (ESI) source, respectively, would be the appropriate analytical technique [16,22,23,43,44,45]. HPLC-ESI-HRMS has the following advantages in metabolomic profiling of Asteraceae natural products: Simpler sample preparation that entailed no derivatization step as required with GCMS; richness of information of metabolites provided by combining accurate mass with retention time or MS/MS fragmentation data; availability of comprehensive commercial (Dictionary of Natural Products (DNP) with 259,859 entries) and *in-house* (e.g., AsterDB [46]) databases allowed fast and easy dereplication; high sensitivity provided a limit of detection at nanogram levels for minor bioactive components; and high selectivity that is very important in studying complex crude extracts [2,21,47,48,49]. The HRMS data allowed accurate dereplication from commercial databases of monoisotopic masses of known natural products while occurrence of isomers can be separated by chromatography. On the other hand, utilizing *in-house* databases has a great advantage in terms of suitability because both reference standards and samples can be analyzed under similar chromatographic conditions and spectrometric parameters. However, co-injection of available reference standards, MS/MS experiments, and identification of isolated pure compounds by nuclear magnetic by NMR (especially for new natural products) have also been utilized as part of the process to confirm structure identity of the bioactive compounds [16,17,22].

Many studies on species from Asteraceae have utilized HPLC-ESI-HRMS for phytochemical studies and/or chemotaxonomic applications [50,51,52,53,54,55,56]. However, only a few studies on Asteraceae metabolome have been performed to find biomarkers of biological properties [28,57,58]. Furthermore, most of the studies evaluated only the metabolome of different extracts from a single or small number of related species to guide discovery of biomarkers and their biological activity [57,58,59,60,61,62,63]. Recently, we employed the J48 decision tree to determine (bio)markers for dual inhibition of COX-1 and 5-LOX from the HRMS metabolite profile data of a diverse set of Asteraceae plant extracts [28]. The decision tree chooses the attributes (X variables) of the data that most effectively split its set of samples into subsets enriched under one class of AI property(Y variable). In this study, we utilized HPLC-ESI-HRMS along with MSA (O2PLS-DA) to also pinpoint dual inhibitors of COX-1 and 5-LOX directly from the crude plant extract. Moreover, O2PLS-DA sorts and simultaneously defines the biomarkers from the X variables for each of the different AI properties (Y variables) according to the VIP (Variable Importance in Projection) scores of the attributes. This strategy has never been applied to study a more diverse set of species originating from different tribes and genera of a huge plant family. For the first time, this metabolomic approach was employed to find biomarkers for a specific biological activity which in parallel also give us a chemotaxonomic insight of the investigated species from Asteraceae. In addition, the chemical profiles of most of these species were obtained for the first time through a sensitive, high-resolution, and comprehensive analytical method.

## 2. Results and Discussion

### 2.1. Chemical Profile (HPLC-ESI-HRMS) of the Extracts

A diverse set of species (*n* = 57) from various tribes with different phylogenetic relationships within the Asteraceae family were chosen for this study (Table 1 and Table S1, [28]) which demonstrated huge diversity of secondary metabolites (Figures S1 and S2) [64,65]. Flavonoids, sesquiterpene lactones, chlorogenic acid congeners and other compounds, that are well-known for their AI potential [30,32], were initially identified from DNP and AsterDB databases through the MZmine software [66] to obtain an overview of the composition of the extracts. Using the monoisotopic exact mass from the databases, identification of the major peaks of well-studied extracts was coherent and comprehensive. As an example, dereplication data for *Tithonia diversifolia* (sample #56) is presented in Table 2. In this study, a total of 6052 peaks (*m*/*z*, RT) were found for the 57 species, and a complete identification of all peaks is neither necessary nor viable. In addition, the presence of putative novel compounds would obviously not be identified from a database. In fact, the coherence achieved in this study was remarkable when compared to most of the earlier studies, where metabolomic profile of evaluated species were just obtained from the same genera or fractions of extracts from single species resulting to quite similar chemistries [57,58,59,60,61,62,63]. The chemical diversity of compounds evaluated in most of the previous studies was much lower than the chemical diversity tackled in this study. It is important to highlight that the detection of compounds of diverse chemistry is fundamental in screening active extracts from different genera prior to determining the type of chemistry that can be associated to the mechanism of action. In this context, the focus of this study was on the dereplication and identification of substances that correlate with the AI property for dual inhibition of COX and LOX (Table 1).

**Figure 1:**
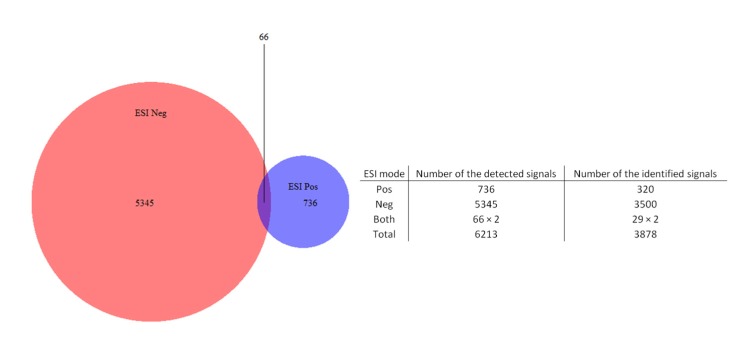
Venn diagram showing the diversity of signals detected in HPLC-ESI-switch mode-HRMS.

The highly diverse chemical profiles of these extracts were managed through suitable data treatment with metabolomics softwares MZmine and SIEVE^®^ before the data was subjected to multivariate statistical analysis [67]. The metabolites’ variability in their physical properties was taken into consideration. These include differences in the capability of the metabolites to ionize either in the positive or negative modes or in both modes as well as their maximum absorbance at different UV wavelengths. Detection of as much metabolites as possible was accomplished both by switch mode-HRMS and UV through a photodiode array (PDA) detector to cover a wavelength range between 220 to 400 nm. Thus, utilizing MS and UV as parallel detectors was important to discern the diversity of the secondary metabolites, as one technique complemented the other. The Venn diagram showed that 87% of the compounds were detected in negative mode and 13% of the compounds were detected in positive mode while only 1% was detected in both modes (Figure 1). The pool of information which consisted of the retention times, UV, and *m*/*z* data was important in the dereplication process (Figure S2 and Table 2). Additionally, the chemical profiles through HPLC-UV-ESI-MS of several of the studied species were revealed for the first time (as exemplified in Table 2) and can be useful for future studies.

**Table 1:** Extracts with dual inhibition activity towards COX-1 and 5-LOX.

Species	Sample Codes	Chemistry Investigated/AI Evidence	Tribe *
*Cichorium intybus* L. (chicory)	19	Yes [37]/Yes [37]	Cichorieae Lam. & DC.
*Minasia scapigera* H. Rob.	40	No/No	Vernonieae Cass.
*Piptolepis monticola* Loeuille	41	No/No	Vernonieae Cass.
*Prestelia eriopus* Sch. Bip.	42	No/No	Vernonieae Cass.
*Solidago microglossa* DC. (arnica do campo)	46	Yes [68]/Yes [68,69]	Astereae Cass.
*Sphagneticola trilobata* (L.) Pruskei	49	Yes [70]/Yes [70]	Heliantheae Cass.
*Tithonia diversifolia* (Hemsl.) A. Gray (tree marigold)	56	Yes [71]/Yes [7]	Heliantheae Cass.
*Vernonia herbacea* (Vell.) Rusby	57	Yes [51]/No	Vernonieae Cass.
*Vernonia platensis* (Spreng.) Less.	58	Yes [51]/No	Vernonieae Cass.
*Vernonia polyanthes* Less. (assapeixe)	59	Yes/Yes [72]	Vernonieae Cass.
*Vernonia rubriramea* Mart. Ex DC.	60	No/No	Vernonieae Cass.
*Viguiera robusta* Gardner	66	Yes [73]/Yes [73]	Heliantheae Cass.
*Viguiera trichophylla* Dusén	67	No/No	Heliantheae Cass.

* According to Funk *et al.* 2009 [64].

**Table 2:** Dereplicationdata of the major compounds of the dual inhibitor extract of *Tithonia diversifolia* (extract code #56).

Peak Area	*m*/*z*	Retention Time	Molecular Formula	UV Peak at Maximum Absorbance (nm)	Dereplication	Literature *
8.50 × 10^7^	153.0195 (M−H)^−^	11.2	C_7_H_6_O_4_	255; 292	protocatechuic acid	[74]
9.12× 10^7^	353.0883 (M−H)^−^	13.9	C_16_H_18_O_9_	300; 323	5-*O*-*E*-caffeoylquinic acid	[7]
7.85× 10^7^	355.1022 (M+H)^+^	13.9	C_16_H_18_O_9_	300; 323	5-*O*-*E*-caffeoylquinic acid	[7]
3.98× 10^8^	515.1200 (M−H)^−^	19.2	C_25_H_24_O_12_	295; 326	3,5-di-*O*-*E*-caffeoylquinic acid	[74]
3.44× 10^7^	517.1340 (M+H)^+^	19.2	C_25_H_24_O_12_	295; 326	5,3-di-*O*-*E*-caffeoylquinic acid	[74]
3.32× 10^8^	515.1200 (M−H)^−^	19.7	C_25_H_24_O_12_	295; 326	4,5-di-*O*-*E*-caffeoylquinic acid	**
2.05× 10^7^	517.1340 (M+H)^+^	19.7	C_25_H_24_O_12_	295; 326	5,4-di-*O*-*E*-caffeoylquinic acid	**
7.29× 10^7^	367.1767 (M−H)^−^	23.9	C_19_H_28_O_7_	211	tagitinin A	[7,71]
2.41× 10^7^	369.1906 (M+H)^+^	23.9	C_19_H_28_O_7_	211	tagitinin A	[7,71]
2.51× 10^7^	349.1644 (M−H)^−^	27.0	C_19_H_24_O_6_	254	tagitinin C	[7,71]
5.14× 10^6^	349.1643 (M−H)^−^	28.4	C_19_H_24_O_6_	210	tagitinin F	[71]

* All hits were found in AsterDB database or DNP. ** Described for the first time in *T. diversifolia*. The identifications were confirmed by the retention times of the standards run in the same chromatographic conditions.

### 2.2. Data Treatment and PCA

The reproducibility of the measurements in this study was demonstrated by the superimposable chromatograms of three replicate injections of a randomly selected sample (extract #33). The sample was injected in the beginning, the middle and at the end of the HPLC-ESI-MS 57-sample sequence experiment (Figure S3). Slight variation in the intensity and peak area of some peaks was also observed. However, with or without data normalization of the entire 57 samples, the data for the three replicates were also found to be similar by PCA analysis (Figure S4). The coefficient of variation (CV = standard deviation/mean) was small with a CV = 0.43 for the datasets in the positive mode and CV = 0.47 in the negative mode. The CV plot (Figure S5) of the three replicate injections for extract #33 afforded a mean CV log ratio < 0.25 for the majority of its attributes in both positive and negative mode. While the CV plot (Figure S5) of all 57 extracts together gave a mean CV log ratio > 0.40 for the majority of their attributes in both modes of ionization. This result is coherent because variation between the different 57 sample extracts was expected and must occur. The significant difference in CV log ratios between the two dataset treatments confirmed reproducibility of the MS data results (Figure S5).

**Figure 2:**
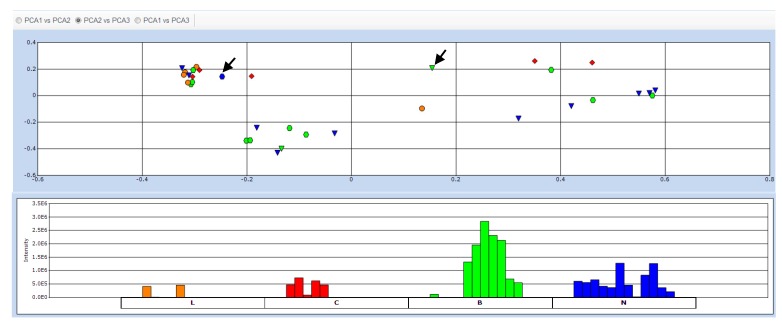
Principal Component Analysis (PCA) of all extracts. The extracts were colored according to their anti-inflammatory activities: Green hexagon (B) for the extracts able to inhibit both enzymes (COX and LOX), orange circle for those able to inhibit only LOX (L), red square for those inhibiting only COX (C),and blue triangle for those found to be inactive (N) against both enzymes. Two reference samples for negative (blue hexagon) and positive (green triangle) activity were indicated by arrows. The trend bar graph indicated the occurrence of a single metabolite in each of the studied extracts.

**Figure 3:**
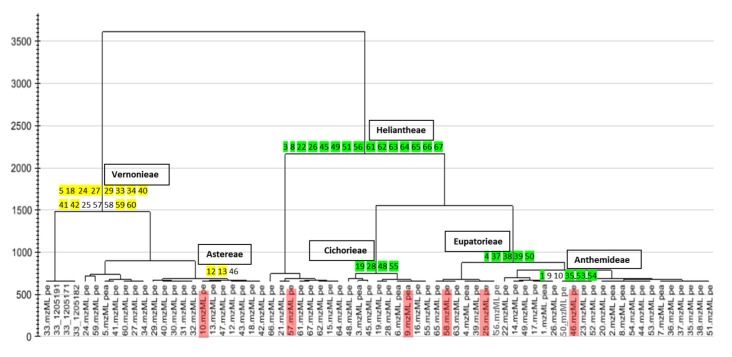
Hierarchical Cluster Analysis (HCA) dendrogram for Asteraceae plant extracts analyzed in this study (*n* = 66, included root, stem and flower parts for some species). Highlighted (green and yellow) numbers represent sample codes of species-related extracts that clustered together in the PCA according to their similarity in the chemistry of their metabolomes. Red highlighted sample codes represent extracts with unique chemistry when compared with other extracts within their respective taxa.

PCA of the HRMS data (Figure 2) that was pre-processed both by MZmine, SIMCA-P + 12.0^®^ and Sieve^®^, disclosed the grouping of the samples according to their phylogenetic proximity [50,51,75]. Extracts prepared from the same genera or tribes were clustered together as illustrated by Hierarchical Cluster Analysis (HCA) of its PCA results (Figure 3). The HCA dendrogram exhibited clear groupings according to their phylogeny but not their anti-inflammatory properties. The phylogenetic grouping was not evident in the PCA scatter plot. Samples were clustered by PCA based on chemical similarity of the extracts. The PCA plots (Figure 2 and Figure S4) showed overlapping samples and groups were not well separated. Thus, it was not easy to define the difference between groups as well as the chemical similarity between respective samples. Interestingly, the HCA dendrogram exhibited significant separation between the Vernonieae and Heliantheae tribes which is comparable to the phylogenetic diagrams of the Asteraceae tribes according to Bremer [76] and Funk *et al.* [64], where both tribes belong to well-separated clades. The HCA results from Simca P+ was validated with Programme R (Figure S6) which even with few samples interfering on the phylogenetic tree, it was possible to see similar results. These results substantiated the use of metabolomics as a potential chemotaxonomical tool [50,51,75]. Nevertheless, unsupervised MSA by PCA did not contribute in the determination of biomarkers for dual inhibition because the samples were not grouped according to their AI properties (Figure 2). However, the trend bar graph of individual attributes (example shown in Figure 2) indicated significant intensity differences of distinct metabolites between AI property classes. This prompted us to apply a suitable supervised MSA to determine these unique biomarkers.

### 2.3. Determination of Biomarkers for Dual Inhibition by O2PLS

**Figure 4:**
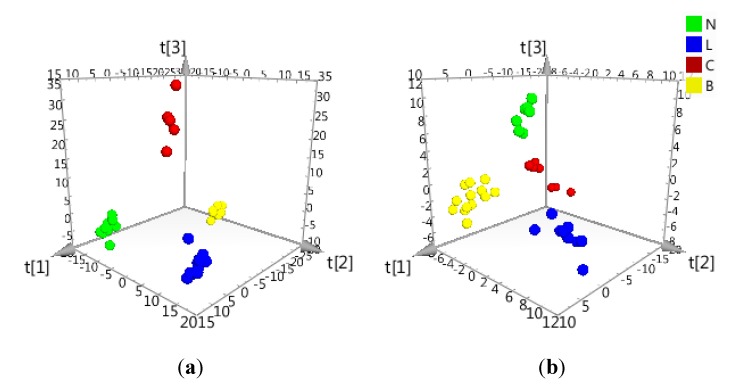
(**a**) negative mode, *R*^2^ = 0.97; (**b**) positive mode, *R*^2^ = 0.92. Three dimensional O2PLS-DAscore plot of the HRMS data for 57 Asteraceae leaf extracts grouped according to their AI properties to inhibit COX-1 and/or 5-LOX. Sample codes are found in Table 1 and Table S1. The sample codes were colored as follows: Dual inhibition = yellow (B); only COX-1 inhibition = red (C); only 5-LOX inhibition = blue (L); and no inhibition = green (N). Sample codes are found in Table 1 and Table S1.

**Figure 5:**
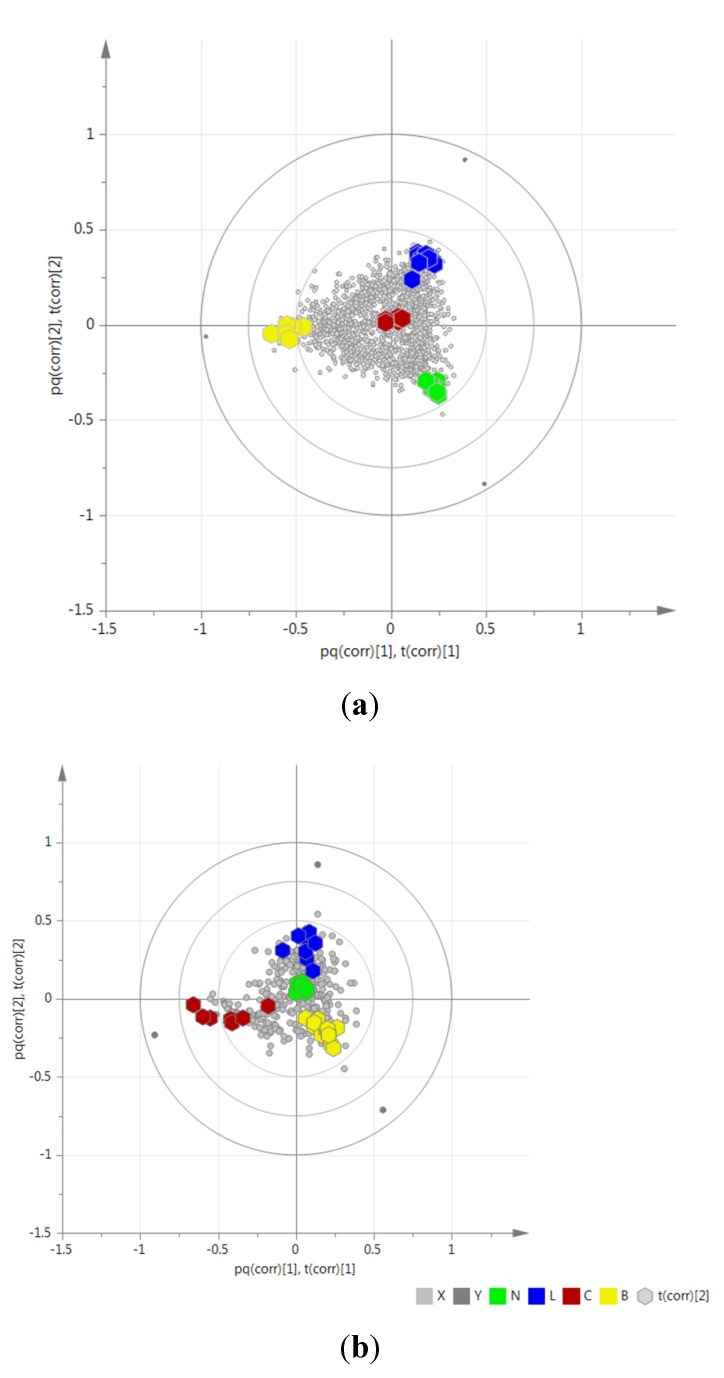
(**a**) Negative mode; (**b**) Positive mode. O2PLS-DA biplots indicating the distribution of the extracts and their metabolome (X-variables) according to their AI property (Y variables).The sample codes were colored as follows: Dual inhibition = yellow (B); only COX-1 inhibition = red (C); only 5-LOX inhibition = blue (L); and no inhibition = green (N). Sample codes are found in Table 1 and Table S1.

O2PLS-DA of the processed HPLC-ESI-HRMS data (Section 2.2.) along with their AI properties found a clear separation among the samples in accordance to their ability to inhibit COX and/or LOX (Figure 4 and Figure 5). The *R*^2^ values were 0.97 and 0.92 for the HPLC-ESI-HRMS negative and positive data, respectively. *R*^2^ values described the amount of Y variables illustrated by the model after cross-validation which gave an overview of the model fitness. *R*^2^ very close to 1.0 was desired, although values >0.5 were considered good values due to the component complexity of the samples [24,77]. However, even for samples with highly complex composition profile (Figures S1 and S2), the *R*^2^ values were >0.9, which indicated a significant statistical difference in chemical composition between groups with different AI properties. This implied that each of the active groups of extracts yielded their respective unique group of metabolites that are not present in the non-active extracts as implicated by the clustering three dimensional O2PLS-DAscore plot (Figure 4).

The compounds correlating with dual inhibition of COX-1 and 5-LOX were targeted and read out from the loading plots. The loading features found at a similar locus to the corresponding extract on the score plot represent the unique (bio)markers for each of the defined AI property as illustrated by a biplot of the score and the loading plots(Figure 5). The VIP scores and correlation coefficients (positive Y-related coefficient) were also considered in identifying the biomarkers for dual inhibition (Table 3). Variables with VIP values > 1 estimated the most important variable in the projection while the correlation coefficients with Y expressed how strong the property is correlated with the variables [63]. For the dual inhibition of COX-1 and 5-LOX and with VIP > 1, nine compounds each were detected from the ESI negative and positive modes (Table 3).

**Table 3:** Biomarkers correlated to dual inhibition of COX-1 and 5-LOX arranged according to their VIP scores.

ID	VIP Scores	*m/z*	RT	Mass Error (ppm)		MF	Hits (Number of Hits from SciFinder/DNP2015/AsterDB) *^a^*
**Negative Mode**							
2054	1.92232	359.0778	14.2	1.656	[M−H]^−^	C_18_H_16_O_8_	F and PC (381/97/6) => Hexahydroxyflavone; TrMe ether, chrysosplenol D ^b^
3913	1.91038	341.0883	12.2	1.410	[M−H]^−^	C_15_H_18_O_9_	PC (162/11/1)
2488	1.89577	679.1505	16.4	3.175	[M−H]^−^	C_30_H_32_O_18_	F (16/2/0)
5001	1.78812	431.1931	18.7	1.912	[M−H]^−^	C_20_H_32_O_10_	PC and other classes (91/0/0)
671	1.71425	487.1256	20.0	2.042	[M−H]^−^	C_24_H_24_O_11_	F and PC (101/14/1) => acacetin-7-*O-*β*-*d-(3′′-acetyl)-glucopyranoside ^c^ [78]
2610 *	1.57931	447.1306	17.5	2.051	[M−H]^−^	C_22_H_24_O_10_	F and PC (231/66/0)
815*	1.38122	509.2246	16.9	1.248	[M−H]^−^	C_22_H_38_O_13_	Saccharides (27/1/0) => β-*D*-glucopyranose, 1-[8-(β-d-glucopyranosyloxy)-2,6-dimethyl-2-octenoate] was found from the DNP
694	1.30532	415.1255	15.6	0.958	[M−H]^−^	C_18_H_24_O_11_	PC (81/15/0)
4582 *	1.29768	470.9873	12.4	1.023	[M−H]^−^	C_23_H_6_O_11_N	(0/0/0)
3144 **	0.957026	513.2714	19.0	1.789	[M−H]^−^	C_26_H_42_O_10_	ST (80/15/0)
829 **	0.834219	411.1797	25.3	1.413	[M+Cl]^−^	C_18_H_34_O_8_	d-glucopyranose,2,3-dihexanoate (107/1/0)
**Positive Mode**							
1273	1.61922	361.1643	32.0	0.774	[M+H]^+^	C_20_H_24_O_6_	STL, D and PC (1850/219/27) ^d^
1282	1.60527	261.1118	32.1	1.077	[M+H]^+^	C_15_H_16_O_4_	STL, PC and F (3723/158/10)
1637 *	1.56815	349.1642	28.2	0.897	[M+H]^+^	C_19_H_24_O_6_	STL, D and PC (1424/164/12), identified as tagitinin F
1116 *	1.54159	600.2654	16.8	0.635	[M+NH_3_]^+^	C_28_H_38_O_13_	(0/0/0)
1190	1.42996	611.1400	20.0	0.717	[M+H]^+^	C_30_H_26_O_14_	F (81/25/0)
1623	1.36987	426.2120	27.9	0.511	[M+NH_3_]^+^	C_21_H_29_O_8_	STL (0/0/0)
1436	1.18345	418.1857	33.9	0.688	[M+NH_3_]^+^	C_22_H_25_O_7_	D, F (6/0/0)
1615	1.16202	363.1799	30.5	0.852	[M+H]^+^	C_20_H_26_O_6_	STL, D and PC (1858/345/12) ^e^
692 *	1.0815	449.1075	19.0	0.607	[M+H]^+^	C_21_H_20_O_11_	F (258/101/2) ex.: Quercetrin occurs in Asteraceae (e.g., in the dual inhibitor extract of *Solidago microglossa* #46 [68]); luteolin, 7-β-d-glucopyranoside is widespread in plants, and also occurs in Asteraceae
1207 **	0.88865	509.1290	20.6	0.160	[M+H]^+^	C_23_H_24_O_13_	F (76/39/0)
1333 **	0.862979	270.1697	29.1	1.112	[M+NH_3_]^+^	C_14_H_20_O_4_	(6048/78/0) Butanoic acid, 3-methyl-,4-(1*R*,2*S*-dihydroxypropyl)phenyl ester
276 **	0.818718	415.2112	34.5	0.634	[M+H]^+^	C_24_H_30_O_6_	STL, D and PC (1438/43/0)

PC: Phenolic compound(s); STL: Sesquiterpene lactone(s); ST: Sesquiterpene(s); F: Flavonoid(s), D: Diterpene(s). ID: Identification number designated by MZmine; RT: Retention time in min; MF: Molecular formula; MW: Molecular weight; * Dual inhibitor biomarkers detected also by J48 decision tree (see ref. 31); ** Dual inhibitor biomarkers detected only by J48 decision tree (see ref. 31); ^a^ Number of hits from the respective databases; ^b^ Described for several species from the Asteraceae family, according to the DNP database; ^c^ For all the hits with the MF = C_24_H_24_O_11_, only acacetin-7*-O*-β-d-(3′′-acetyl)-glucopyranoside has been previously described from the Asteraceae family; ^d^ Two different STLs previously described for respective genera *Vernonia* and *Viguiera* (DNP and AsterDB databases); ^e^ STLs previously described for *Tithonia* *diversifolia* and *Viguiera robusta* (DNP and AsterDB databases).

**Figure 6:**
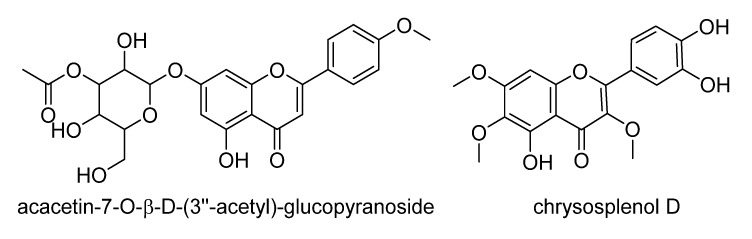
Examples of phenolic compounds dereplicated from Asteraceae extracts with dual inhibition property against COX-1 and 5-LOX.

With the data provided by HPLC-UV-ESI-HRMS, the identification of the biomarkers was not as straightforward as the dereplication of the major compounds (Table 2) from the extracts. Due to the weak MS and UV responses of biomarkers, it is more likely that these metabolites were present as minor components in the extracts (Table S1). As presented in Table 3, by using the predicted molecular formulae, a huge number of isomers gave matching hits from SciFinder and DNP databases. However, special attention was given to compounds isolated within the Asteraceae family or within its genera. In the negative ESI mode, hits consisted mostly of phenolic and flavonoid compounds (Table 3, Figure 6), which have been previously described inseveral species within the family, and in these conditions, their peaks were observed between 15 to 20 min. The MF of C_24_H_24_O_11_ (ID#2054),detected in the ESI negative mode, matched acacetin-7-*O*-β-d-(3′′-acetyl)-glucopyranoside and was the only hit associated with the Asteraceae family [78]. This flavone glucoside was earlier isolated from the flowers of *Chrysanthemum sinense* Sabine and was reported to exhibit significant xanthine oxidase inhibitory activity. Interestingly, the kinetic study indicated a competitive-type of inhibition while at the same time acacetin-7-*O*-β-d-(3′′-acetyl)-glucopyranoside showed a mixed-type inhibition. Flavonoids are known to potentially deter inflammatory pathways [30,31,79] but none of these compounds have been described to specifically inhibit COX-1 and 5-LOX.

**Figure 7:**
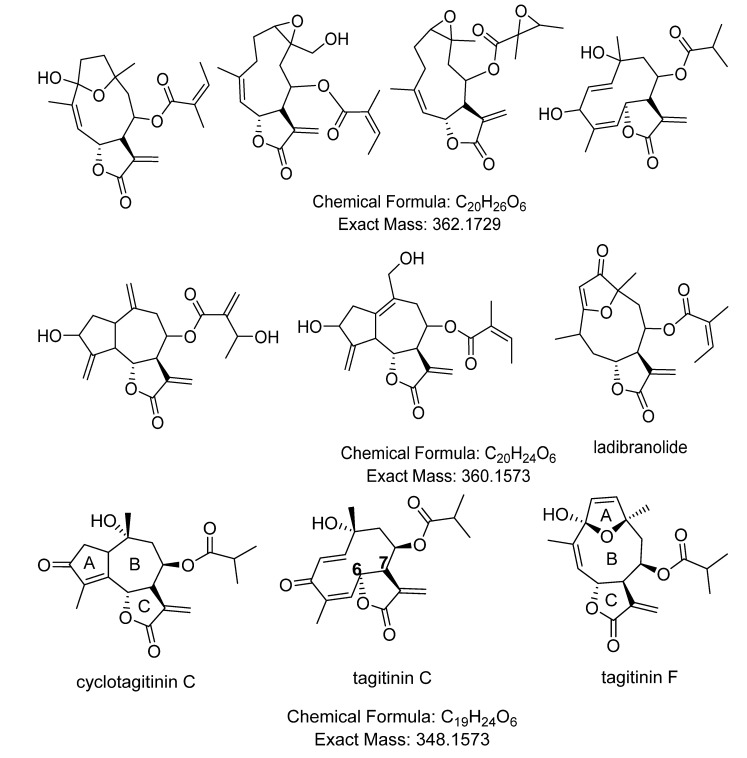
Some sesquiterpene lactones dereplicated from extracts with dual inhibition property against COX-1 and 5-LOX (stereochemistry was not shown due to the possibility of occurrence of isomers/epimers, except for tagitinin F which was confirmed through co-elution and MS/MS fragmentation of a reference standard; Figure 8). Although it should be taken into account that all STLs from the tribes investigated herein (and those shown below) must have α,β orientation at C6/C7.

**Figure 8:**
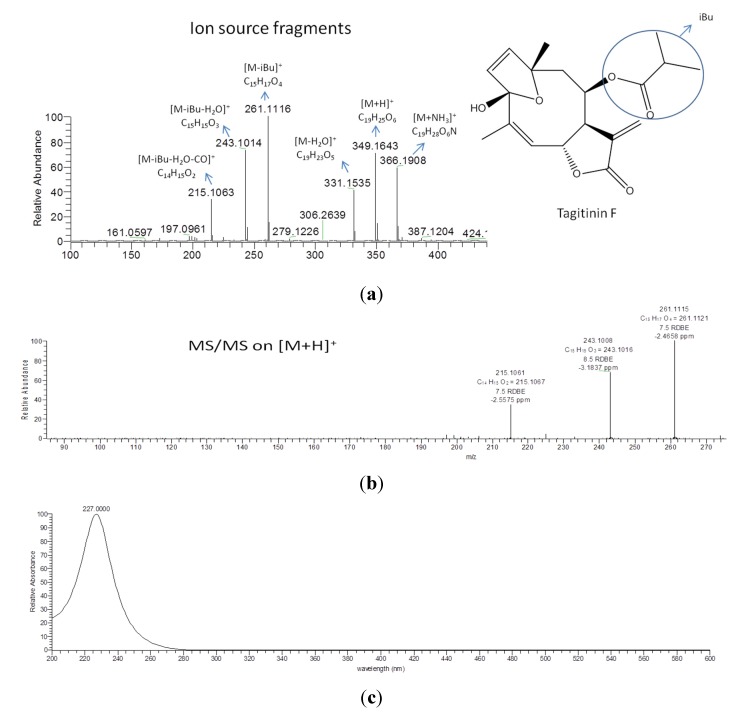
(**a**) The ion source fragments of the standard of tagitinin F; and (**b**) the fragments of MS/MS on [M+H]^+^ on the HPLC-ESI-HRMS analysis are shown. The peaks with the same retention time of the tagitinin F (28.2 min) in the dual inhibitor extracts #40–42, 49, 56, 59 and 60 (Table 1) yielded anion peak at *m*/*z* 349.1643 [M+H]^+^ and gave a similar MS/MS fragmentation pattern as tagitinin F. The peaks also have the same UV spectra as tagitinin F; (**c**). Co-injection confirmed the exact same retention time. These data confirmed the identification of the peaks in the dual inhibitor extracts as tagitinin F.

The positive mode revealed the presence of sesquiterpene lactones (STLs) and/or diterpenes that are relatively more non-polar eluting between 20 and 35 min (Table 3). Hits included STLs previously isolated from *Tithonia diversifolia* and *Viguiera robusta* as well as three different STLs described for the genera *Viguiera* and *Vernonia* (Table 3, Figure 7)*.* *T. diversifolia* and *V. robusta* were among the extracts that exhibited dual inhibition against the enzymes COX-1 and 5-LOX. The presence of tagitinin F, which was earlier isolated from *T. diversifolia*, was then verified by co-elution and MS/MS fragmentation analysis (Figure 7 and Figure 8). The common peak eluting at 28.2 min in the dual inhibitor extracts #40–42, 49, 56, 59 and 60 (Table 1) yielded anion peak at *m*/*z* 349.1643 [M+H]^+^ and gave a similar MS/MS fragmentation pattern.MS/MS ion peaks were observed at *m*/*z* 331.1643 [M−H_2_O]^+^; 261.1116 [M−iBu]^+^; 243.1014[M−iBu−H_2_O]^+^; and 215.1063 [M−iBu−H_2_O−CO]^+^, which confirmed the identity of the peak (ID#1637) to be tagitinin F (Figure 8). Tagitinin F was evaluated for the first time and also showed to be a dual inhibitor of COX-1 and 5-LOX with IC_50_ values of 0.001 and 18.5 µM, respectively. It is worth mentioning that tagitinin F (ID#1637, Table 3) was also detected as a dual inhibitor of COX-1 and 5-LOX using the J48 decision tree classifier [28]. It should be emphasized that tagitinin F is not the major compound in leaves of *T. diversifolia* [7].These findings reveal the quality of our developed metabolomics-based approaches to detect biologically active compounds in extracts.

Sesquiterpene lactones have been patented as potential NSAIDs [80] due to their capability to inhibit the expression of COX-2 in *ex vivo* experiments on inflammatory cells. They are known to inhibit several biological effects aggravated by PLA2 (phospholipase A2) and NF-κB (nuclear factor κB) activation [81,82]. This includes the release of arachidonic acid through inhibition of the PLA2 or expression of enzymes like COX-2, and mediators involved in inflammation through inhibition of the NFκB release to consequently decrease the pro-inflammatory products [81,82]. There have been few studies on COX-1 and 5-LOX inhibition by STL and most of them used cell or other enzymes involved in the inflammatory cascade. Those that evaluated directly on the enzymes found negative results [83]. Rungeler *et al.* studied three STLs and based on previous studies, they generalized that STLs do not act on COX-1 although some derivatives can inhibit PLA2 [83]. The alkylant moiety (α-methylene-γ-lactone group and an α,β- or α,β,γ,δ-unsaturated carbonyl group) is usually responsible for the AI property of STLs. However, other structure requisites are also necessary for a STL congener to display a certain or further mechanism of action on different inflammatory pathways, [82,84], that includes other fine-tuning enzyme activities.

Kinetic studies have shown that STLs tend to inhibit PLA2 non-competitively with the presence of a substrate. The most active STLs in the inhibition of edema-inducing activity, enzymatic activities and myotoxic activity are provoked by PLA2. Through *ab initio* quantum calculations and chemometric methods on the activity of eight STLs in PLA2, Da Silva’s group [81] illustrated how HOMO (Highest Occupied Molecular Orbital) energy, log P, and molecular volume could be accountable for the differences between the most and the less active congeners. In active derivatives, HOMO energy values is low and would favor electron transfer, which is affected by the orientation of the carbonyl group in ring C, *i.e.*, in the γ-lactone ring (Figure 7). A congener with a five-membered ring A can increase its inhibitory property when a transfer charge complex can be formed between PLA2 and the carbonyl group in ring C. The correct position of the carbonyl group in ring C can be achieved with a six-membered ring B. On the other hand, a seven-membered ring shifts the correct positioning of the carbonyl group that could decrease bioactivity. While in tagitinin C, where ring A is absent, the congener was only active against COX-1 with an IC_50_ of 30.1 µM, the STL tagitinin F, with its nine-membered ring B, was found to be a dual inhibitor. A similar structural constraint may also be vitalto achieve dual inhibition of COX-1 and 5-LOX which to date still needs to be studied further. Nevertheless, it should be also taken into account that all eight earlier tested STLs in the cited study did not have the α,β-unsaturated carbonyl with an exocyclic methylene in the γ-lactone ring. This unsaturation is essential for the alkylating property and consequently the biological activity of most STLs[82] such as those from *Tithonia* and *Viguiera*.

Although we could confirm the identity of one biomarker (ID#1637) for dual inhibition of COX-1 and 5-LOX to be tagitinin F, we also must consider the unidentified biomarkers detected in the ESI negative mode that includes the phenolics and flavonoids (Table 3). They were determined in a well-fitted model (Figure 4, Table 4) and are predicted to be potent dual inhibitors as tagitinin F proved to be. The dual inhibitors determined by the decision tree classifier did correlate well with the variables classified under the same group in O2PLS. However, due to algorithm differences and distinct variable selection methods, the identified dual inhibitors by O2PLS listed in Table 3 and those earlier enumerated using the J48 decision tree were comparable but not identical [28]. Not all X variables correlating with a particular AI property are considered by the decision tree, but only those that are able to split the classes according to the Y variables are taken into account [28]. This splitting process is strongly affected by the ion peak intensities of the attributes. The detected biomarkers for dual inhibition from both the decision tree classifier [28] and O2PLS gave VIP scores > 1 (Table 3). Components ID #3144, 829, 1207, 1333, and 276 were only determined as dual inhibitors by the decision tree classifier [28]. These latter components had VIP scores < 1 but > 0.8. With O2PLS, all X variables are accounted for and assigned VIP scores in the selection process regardless the peak intensities of the attributes. Some of the detected biomarkers were not available in the *in-house* database AsterDB and this included the components ID#694, 2488, and 5001 in the negative mode; while in the positive mode, there were ID#1190, 1436, and 1623. One unknown compound (C_20_H_32_O_10_) was found by O2PLS while two unidentified compounds (C_23_H_6_O_11_N and C_28_H_38_O_13_) were uncovered by the decision tree classifier. Except for certain amides, nitrogen compounds are very rare in Asteraceae. These new active natural products can then be targeted for future isolation work.

**Figure 9:**
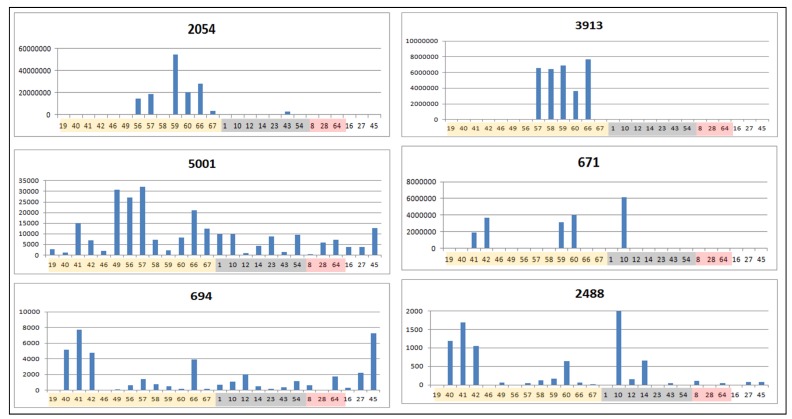
Trend bar graphs indicating the peak areas of some of the biomarkers of dual inhibition of COX-1 and 5-LOX (negative mode HPLC-ESI-HRMS). The graphs are arranged in the order of decreasing correlation values. Extracts were colored as follows: Yellow for dual inhibitors, white for COX-1, grey for non-inhibitors, and pink for 5-LOX inhibitors (Table 1).*p* < 0.05 in *t*-test when comparing the peak area of the biomarkers in the active samples with those from non-dual inhibitors.

**Figure 10:**
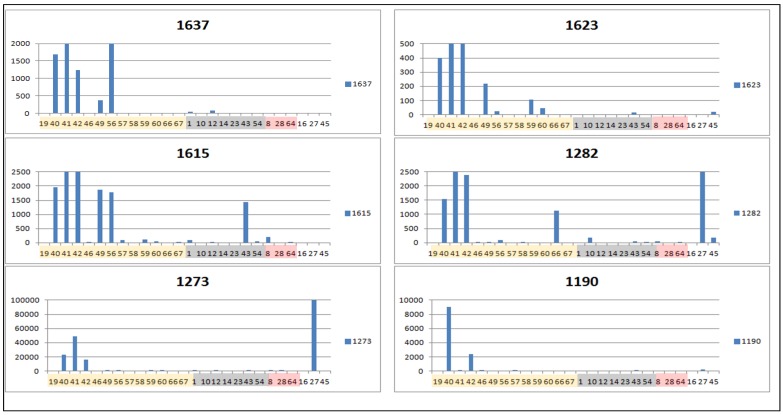
Trend bar graphs indicating the peak areas of some of the biomarkers of dual inhibition of COX-1 and 5-LOX (positive mode HPLC-ESI-HRMS). The graphs are arranged in the order of decreasing correlation values. The graph for biomarker #1436 was omitted because it was quite similar to the graph of biomarker#1273 (Table S1). Extracts were colored as follows: Yellow for dual inhibitors, white for COX-1, grey for non-inhibitors, and pink for 5-LOX inhibitors (Table 1).

The dual inhibition biomarkers with the highest VIP scores (#2054 and #3913 detected in negative mode, #1637 and #1623 detected in positive mode) were found at higher concentrations only in dual inhibitor extracts (Figure 9 and Figure 10). However, the active samples do not have all these biomarkers together (Figure 9 and Figure 10).This may suggest that each of these compounds are single-handedly responsible for the dual inhibition of COX-1 and 5-LOX, and that the respective bioactive compounds do not depend on each other to be more or less active. In fact, we proved that the biomarker #1637 (tagitinin F), determined in two different approaches, is in fact able to inhibit both enzymes *in vitro*. Some of the biomarkers were also found to occur in extracts belonging to other AI property classes defined in this study. As shown in Figure 9 and Figure 10, the intensity of the biomarkers for dual inhibition was generally higher when compared to their incidence in inactive extracts. The occurrence of phenolic and flavonoid biomarkers (Table 3) was more dispersed throughout the different AI property classes, particularly for components ID#694, 2488, and 5001.

### 2.4. Validation of the O2PLS Model for the Predictions of Dual Inhibitors of COX-1 and 5-LOX

The O2PLS-DA model was validated for its prediction of dual inhibitors of COX-1 and 5-LOX from the metabolomic data of the extracts acquired through HPLC-ESI-HRMS. The values obtained from cross validation (*Q*^2^) and external validations (*P*^2^) were > 0.5 (Table 4), indicating that the ability of the model to predict the dual inhibitors was strong [24,77]. The *R*^2^ values were equal to 1, which meant that the O2PLS-DA model was well-fitted. The error values (Root-Mean-Square Error of Cross-Validation-RMSECV) were very low as well (Table 4) and all the extracts predicted as active were in fact dual inhibitors, thus the models had 0% false positive results. The validation using the 3rd test group corroborated this. Samples grouped as single inhibitors of 5-LOX or COX-1 (classes not included in the training group) were not predicted as dual inhibitors, but were rather predicted as inactive, as they should be. However, there were false negative results, in which active extracts were predicted as inactive. This was reflected by *Q*^2^ scores of 0.84 and 0.70 for the positive and negative modes, respectively. Yet these *Q*^2^ scores are considered good as they are >0.6 for external validations (Table 4).

Therefore, through O2PLS-DA, it is easy to distinguish extracts with dual or single inhibition of COX-1 or 5-LOX from the inactive extracts. With assurance, the validation results also verified the O2PLS-DA model in its ability to determine the biomarkers for dual inhibition. The fact that the biomarkers were not the major compounds, made it clear that using classical approaches in natural products research to find these biomarkers would be quite difficult and time-consuming. The application of suitable and sensitive techniques in combination with proper MSA method was fundamental to determine the biomarkers of dual inhibition.

**Table 4:** Prediction model of O2PLS-DA built with HPLC-ESI-HRMS data.

	MS Mode	*R*^2^	*Q*^2^	RMSECV	*P*^2^-group 1 **	*P*^2^-group 2 **	*P*^2^-group 3 ***
**O2PLS-DA**	Negative mode	1.00	0.84	0.19	0.70	0.80	0.80
	Positive mode	1.00	0.70	0.26	0.60	0.60	0.60

* Training group was composed of the following extracts: No activity (n): 1, 43, 10, 12, 14, 23, 54 and dual inhibitors (b): 40, 41, 56, 57; ** Validation groups were composed of the following extracts not included in the training of the model: Group 1-(n): 13, 15, 26, 25, 53, 52 and (b): 66, 67, 49, 46; Group 2-(n): 13, 25 and (b): 58, 60, 19, 42 46; *** Validation group 3 was composed of the following extracts not included in the training of the model plus additional samples with anti-inflammatory properties that were not included in the model training (inhibitors only of 5-LOX (l) or only COX-1 (c) to check if there were false positive results). Group 3-(n): 13, 15, 25; (l): 9, 29, 22; (c): 24; (b): 19, 42, 46; *Q*^2^: Cross validation; *P*^2^: External validation.

## 3. Experimental Section

### 3.1. Plant Material

Leaves from 57 Asteraceae species were either commercially obtained, or previously collected, or received as donations. All information is summarized in Table S1. For species #2, #8 and #53, the inflorescences, not the leaves, were the plant parts that correlated with the anti-inflammatory activity. Because of this, we studied the available inflorescences of these species instead their leaf parts.

The species included in this study were separated into three groups: Food plants, plants known as AI and those unknown as AI. Species from different tribes within the Asteraceae family were chosen for the study (Table S1). This taxonomic diversity led to substantial metabolome diversity.

Purchased plant material has documents to attest their authenticity (#2 Sítio da Mata, Cajuru-SP; #3-Market, Belém-PA; 8#-Santos Flora, São Paulo-SP; #50-Sítio da Mata, Cajuru-SP; #4, #6, #9, #10, #12, 19#, #20, #35, #37, #53, #55, #59, #60-Sítio Irmas Maries, Jardinópolis-SP). Donated materials have voucher specimens deposited in the SPF herbarium at the Department of Botany, Institute of Biosciences, University of São Paulo (USP), São Paulo-SP, Brazil. Their respective voucher specimens are as follows: Species code #1-A.M.S. Pereira 1428; #5-Loeuille *et al.* 537; #13-A.M.S. Pereira 1426; #14-A.M.S. Pereira 1424; #15-M. Nogueira e L.E. Gregório 35; #1 -A.M.S. Pereira 1430; #18-Lusa *et* *al.* 63; #21-DA Chagas-Paula 10; #22-A.M.S. Pereira 1425; #23-DA Chagas-Paula 06; #24-Loeuille *et al.* 531; #25-A.M.S. Pereira 1418; #26-DA Chagas-Paula 09; #27-Lusa *et al.* 61; #28-D.A. Chagas-Paula 08; #29-Loeuille *et al.* 530; #33-Sítio Irmas Maries, Jardinópolis-SP; #34-Loeuille *et al.* 528; #38-A.M.S. Pereira 1400; #39-A.M.S. Pereira 1419; #40-Loeuille *et al.* 529; # 41-Lusa *et al.* 62; #42-Loeuille *et al.* 524; #43-A.M.S. Pereira 1427; #45-A.M.S. Pereira 1422; #46-A.M.S. Pereira 1421; #48-A.M.S. Pereira 1429; #49-DA Chagas-Paula 05; #51-DA Chagas-Paula 07; #54-A.M.S. Pereira 1420; #56-A.M.S. Pereira 1423; #57-ESA 94148; #58 - ESA 94146; #61-M Magenta *et al.* 275; #62-M Magenta *et al.* 440; #63-M Magenta *et al.* 307; #64-AB Bombo *et al.* 56; #65-AB Bombo *et al.* 62; #66-M Magenta *et al.* 454; #67-AB Bombo *et al.* 51. Their identifications were carried out by the persons whose name abbreviations are listed above. Genetic Heritage Management Council authorization number: 010055/2012-6.

### 3.2. Extraction of Compounds from Plant Material

Dried plant material was ground into powder using an analytical mill (A11, IKA^®^). Particle size was below 0.42 mm. Twenty milligrams of each plant powder were extracted with 2 mL of EtOH-H_2_O (7:3) in an orbital shaker (110 rpm and 30 °C) for 24 h. The extracts were further partitioned with *n*-hexane to remove fatty acids, filtered through a 0.2 μm PTFE membrane (Millipore^©^) and dried under reduced pressure. The same extracts were used for UHPLC-HRFTMS analysis and AI assays. This procedure was previously ascertained to extract a high diversity of potentially anti-inflammatory active compounds with high to intermediate polarity [29].

### 3.3. Chemicals and Materials

HPLC-grade acetonitrile (ACN) was purchased from Fisher Scientific, Loughborough, UK. HPLC-grade water was produced by a Direct-Q 3 Ultrapure Water System (Millipore, Feltham, UK). AnalaR-grade formic acid (98%) was obtained from BDH-Merck, Poole Dorset, UK.

### 3.4. HPLC-ESI-HRMS Analysis

The extracts were analyzed by HPLC-ESI-HRMS using a Thermo Scientific Exactive™ equipment (Bremen, Germany) powered by Orbitrap™ technology. The equipment also utilized a Thermo Scientific DAD detector. The analyses were carried out using a C-18 column (ACE^®^)-150 × 3 mm, 3 µm particle size at 30 °C, a flow rate of 0.3 mL/min at gradient elution. The solvent gradient of ACN (A) in H_2_O and 0.1% formic acid (B) commenced with 5% of A in 5 min followed by 5%–100% of A in 50 min and finishing with 100% of A for 10 min. The sample volume injected was 10 μL of a solution of 1 mg of extract/mL in water maintained at 12 °C[28].

The spectrometer was operated in polarity switching mode with the following settings: Scan range 75–1200 *m*/*z*, fragmentation HCD gas off, high resolution, microscan 1, lock mass positive, AGC target balanced, maximum inject time 250 ms. The settings for ESI source were: Sheath gas flow rate 50, auxiliary gas flow rate 17, sweep gas flow rate 0, spray voltage (|kV|) 4.5, spray current (MA) 1.4, capillary temperature (°C) 320, capillary voltage (|V|) 30, tube lens voltage (|V|) 90, skimmer voltage (|V|) 20 [28].

Mass calibration was performed for each polarity prior to analysis and the mass range was extended from 150 to 1500 Da to cover small molecular weight metabolites by inclusion of low-mass contaminants with the standard Thermo calmix masses. Lock-mass correction was also applied to each analytical run using ubiquitous low-mass contaminants to enhance calibration stability [28].

The data was recorded and processed using Xcalibur 2.1.0 software package (ThermoScientific^©^). The samples were randomly analyzed with one blank, while additional injections (3 times) were done on one sample in the beginning, middle and at the end of the sequence to control ionization and retention time reproducibility.

### 3.5. Data Treatment

The mass spectral raw data (along with the retention time - RT) of all samples were sliced to positive and negative datasets using the MassConvert tool from ProteoWizard [85] and then converted to the .mzML format prior to importing to MZmine 2.10 for data treatment. MZmine [66] is an open source software used to perform deconvolution (threshold: 5%; RT: Range 12 s; min. relative height: 15%), deisotoping (*m*/*z* tolerance: 0.001; RT tolerance: 6s), alignment (join aligner *m*/*z* tolerance: 1 ppm; RT tolerance: 30 s; weight for *m*/*z*: 15 and for RT: 10), gap filling (intensity, *m*/*z* and RT tolerance respectively: 1%; 0.001 *m*/*z* and 30 s) and dereplication [67,86,87]. After removing a few non-peak-shape features by visual inspection, both data sets were exported from MZmine as csv files for further statistical analysis. These files contained the identification number, *m/z*, retention time and area of each peak.

The mass spectral raw data was also processed with Sieve 2.0.180^®^ (Thermo Scientific^©^) using the default parameters. This software works in a very similar way to MZmine.

### 3.6. MSA

The processed data sets were then analyzed both by unsupervised (PCA) and supervised (O2PLS-DA™) multivariate methods with unit variance data scaling using the SIMCA-P+ 12.0 software (Umetrics, Umeå, Sweden). The same statistical parameters were used for both methods. The model for prediction for the data was also validated. Some sample data were used to train the O2PLS-DA model while another group of data was used to test the model (Table 4).The samples were randomly grouped where 70% of the samples were assigned to the training group and 30% to the test group for validation. This proportion of 70:30 was considered for each of the AI classes of dual and non-dual inhibitors. Thus, the 70% included both dual and non-dual inhibitors to build the model. The remaining 30% was used for the test group to validate the model. The 70:30 proportion has been earlier validated for model prediction [28]. The HCA were performed in RStudio version 0.98.1102^©^.

### 3.7. Dereplication

Dereplication [67,86,87] was performed with the *in-house* database AsterDB, which contains only substances described from species of the family Asteraceae, and the commercially available Dictionary of Natural Products^©^ (DNP). The dereplication was also confirmed with SciFinder^®^ and by literature search.

## 4. Conclusions

A sensitive analytical technique (HPLC-ESI-HRMS) associated with a suitable multivariate statistical analysis (O2PLS-DA) used in this study allowed the determination of biomarkers of dual inhibition of cyclooxygenase and lipoxygenase in complex plant samples. The limitation of this strategy is the unsuccessful dereplication of isomeric compounds based only on their mass spectral data while the UV’s limit of detection was not good enough to perceive components at nanogram levels. The identity of an isomer could only be verified by co-elution and/or MS/MS experiments. However, sometimes epimers are not distinguishable only by MS/MS and they are very common in Asteraceae, especially at the orientation of the side chain ester. Unidentified biomarkers can be used for retrieving new active extracts from the plant family. The validated O2PLS models predicted active extracts from the provided chemical information. For the first time this approach was applied to such complex diverse samples obtained from a wider range of different species, genera and tribes like the Asteraceae family, and thus resulting to a significant statistical analysis. The main advantage of the strategy was the detection of the biomarkers as minor compounds at micro- or nanogram levels without any time-consuming isolation step.

Additionally, the approach can also be employed in the discovery of new or lead biomarkers, like tagitinin F, to target a mechanism of action before commencing a huge isolation project. New biomarkers will potentially provide compounds with interesting pharmacological characteristics. The metabolomic strategy applied herein precludes the redundant discovery of known compounds with common mechanisms of action like that of a classical phytochemical approach, thus solving one of the main drawbacks of natural products research. Our approach can thus be applied in drug discovery in general, such as helping to find new medicines, nutraceuticals, as well as agrochemicals in plants.

## Supplementary Files

Supplementary File 1
