# Supplementary material for: A Metabolomic Approach to Target Compounds from the Asteraceae Family for Dual COX and LOX Inhibition
Source: Metabolites. 2015 Jul 8;5(3):404–30. doi: 10.3390/metabo5030404 (PMC4588803; doi:10.3390/metabo5030404)
Supplement: Supplementary File 1 [file metabolites-05-00404-s001.pdf]

Supplementary Material

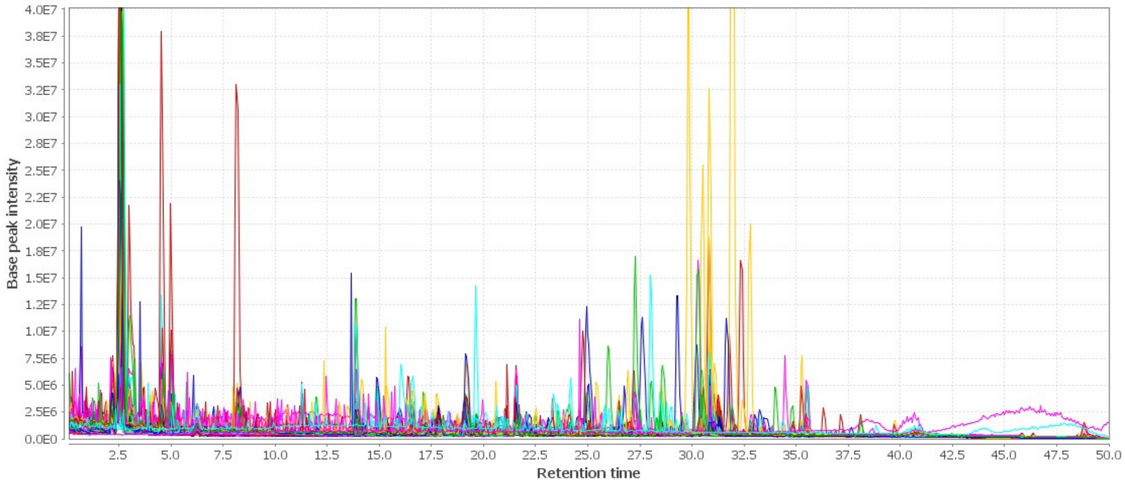

(**A**)

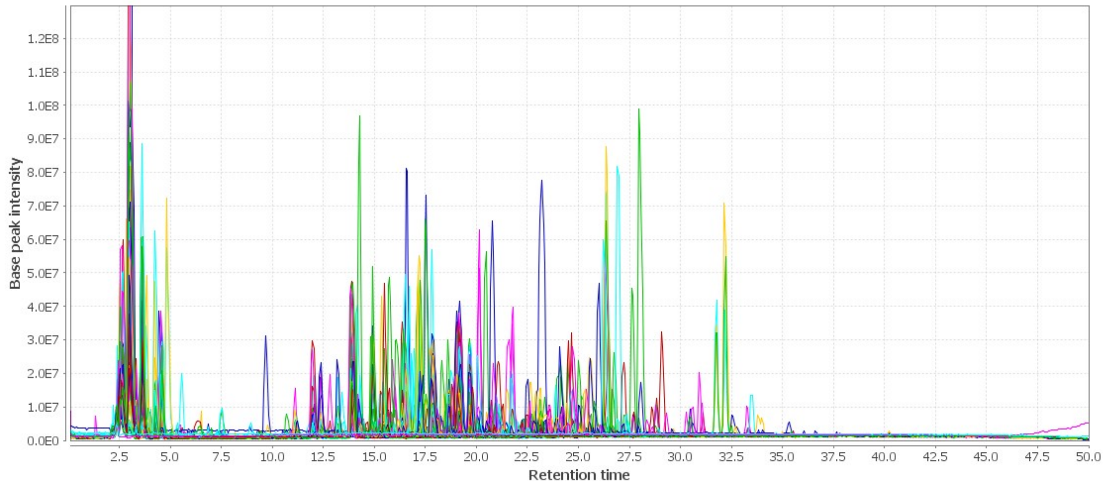

(**B**)

Figure S1. Overlap of all the HPLC-HRFTMS chromatograms visualized on the MZmine software. The chromatograms were detected in positive (**A**) and negative (**B**) modes.

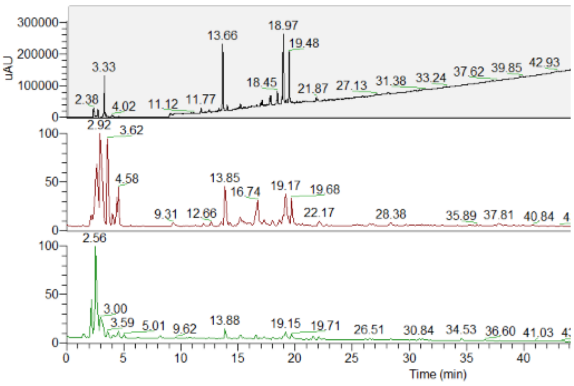

(**1**)

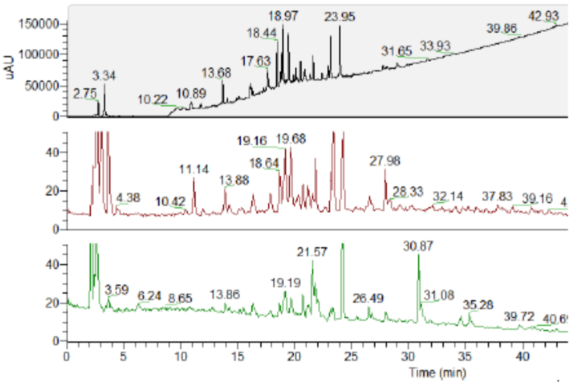

(**2**)

Figure S2. *Cont.*

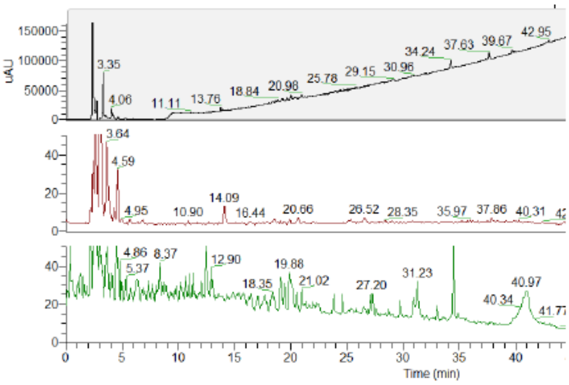

(3)

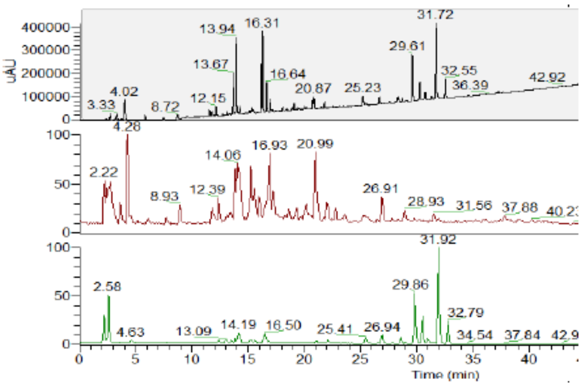

(4)

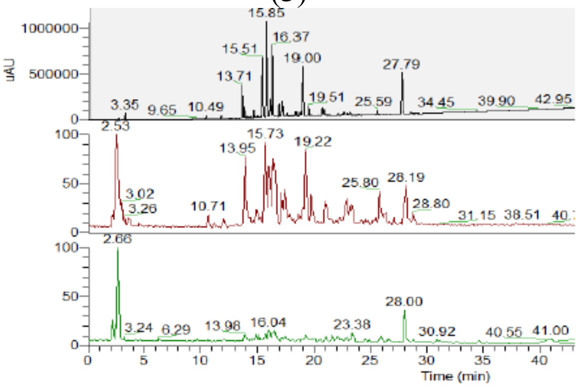

(5)

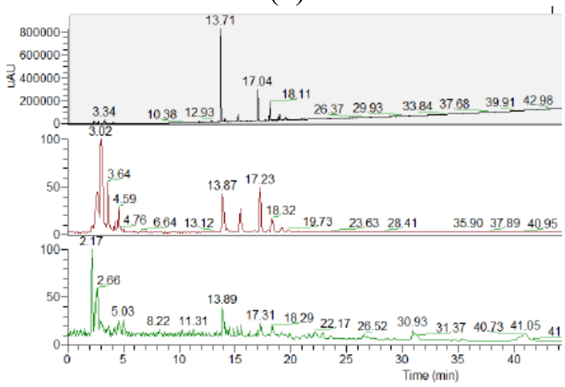

(6)

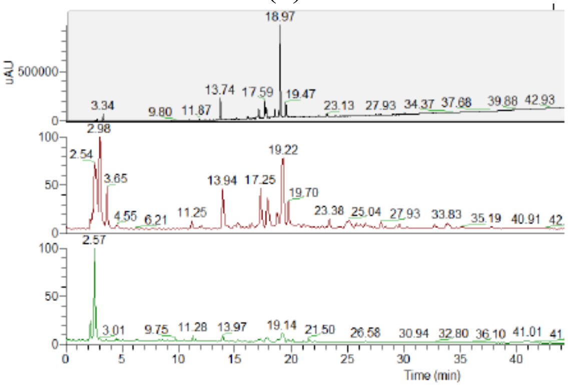

(7)

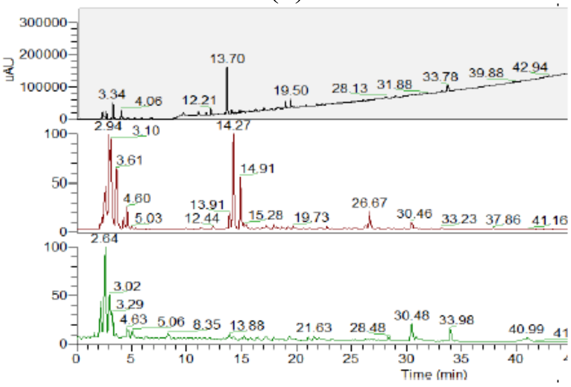

(8)

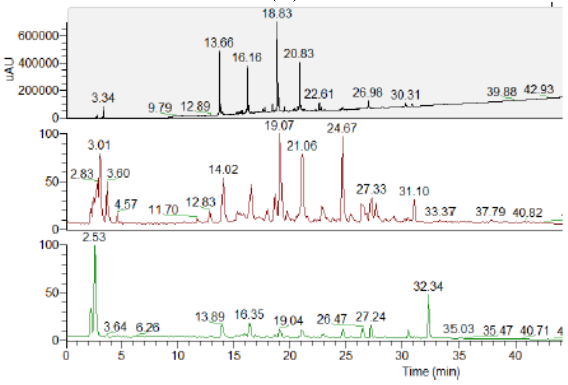

(9)

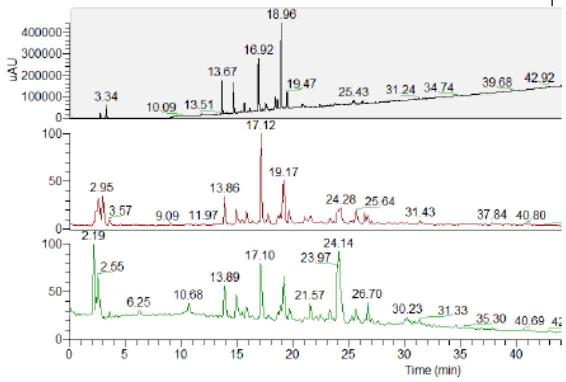

(10)

Figure S2. *Cont.*

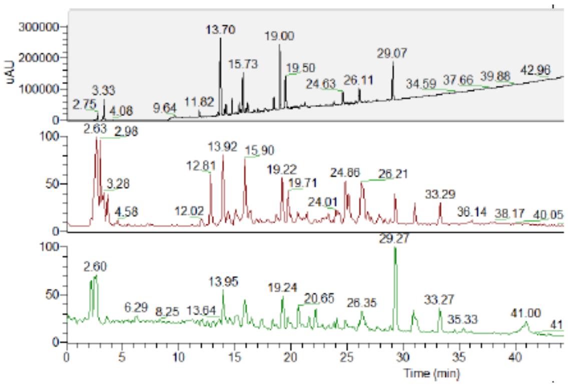

(11)

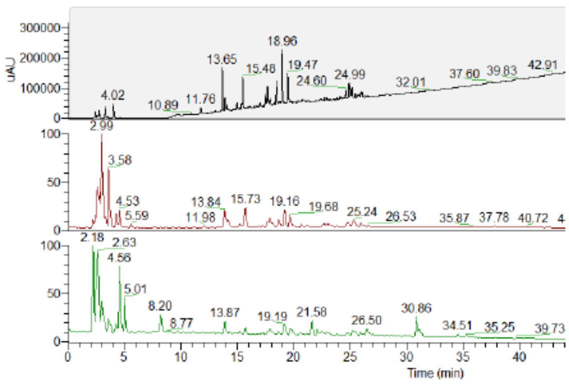

(12)

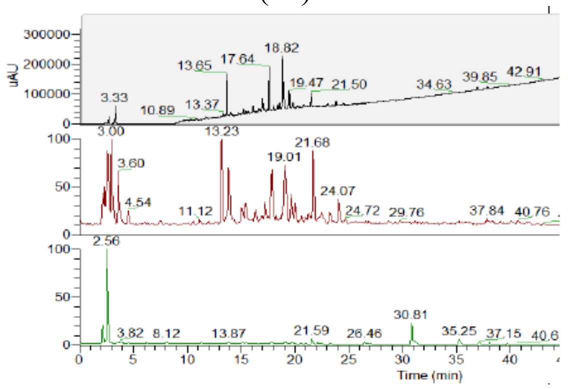

(13)

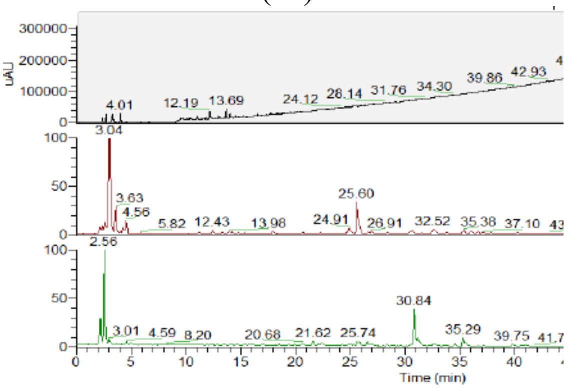

(14)

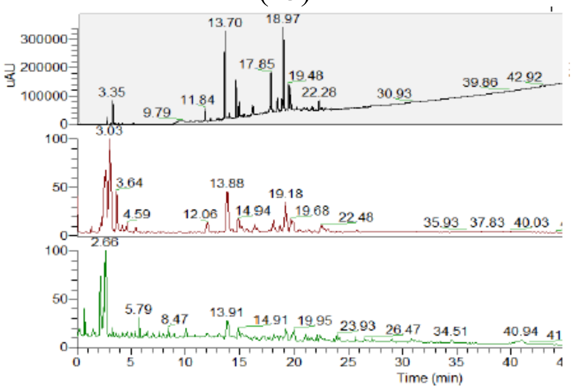

(15)

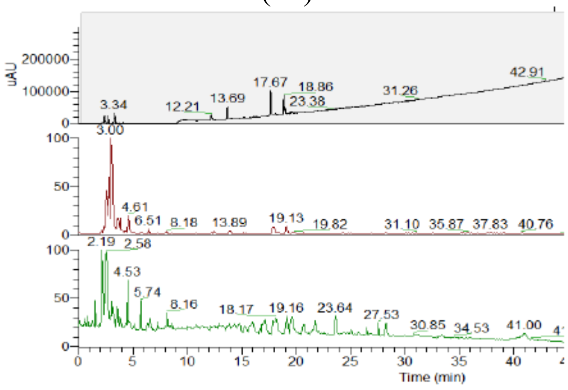

(16)

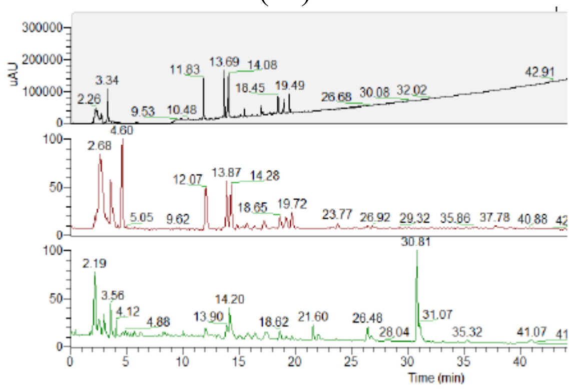

(17)

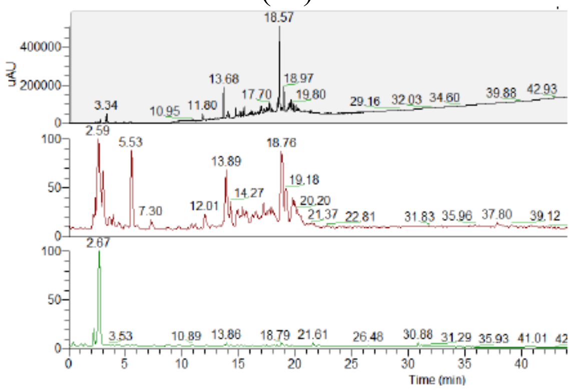

(18)

Figure S2. *Cont.*

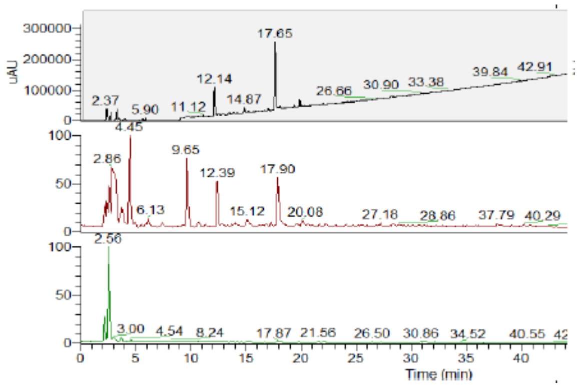

(19)

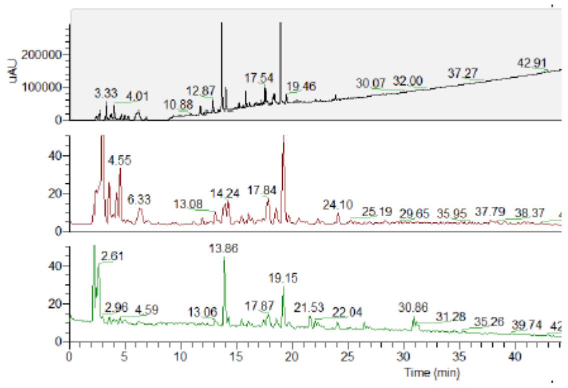

(20)

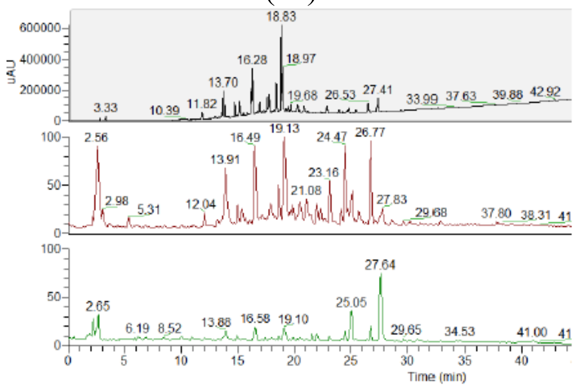

(21)

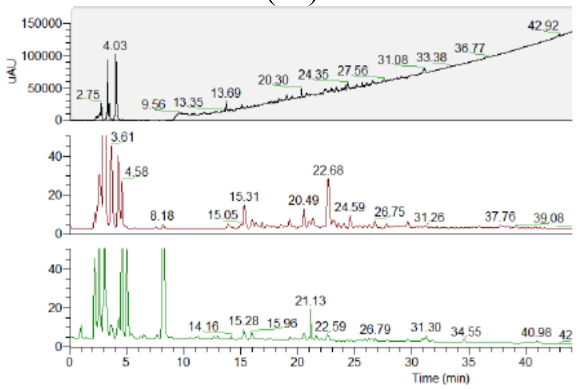

(22)

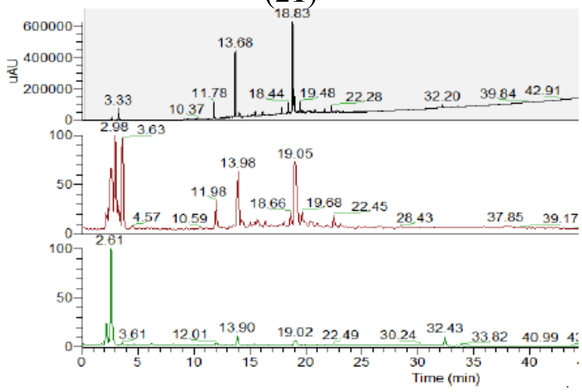

(23)

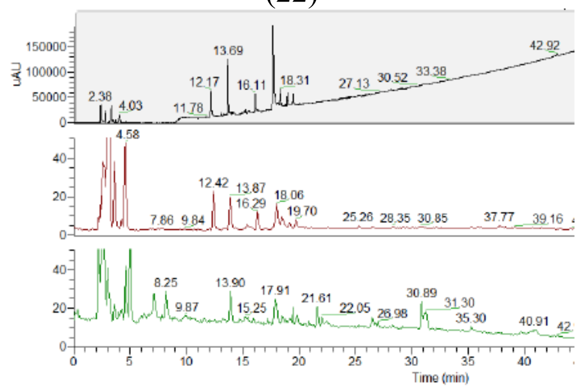

(24)

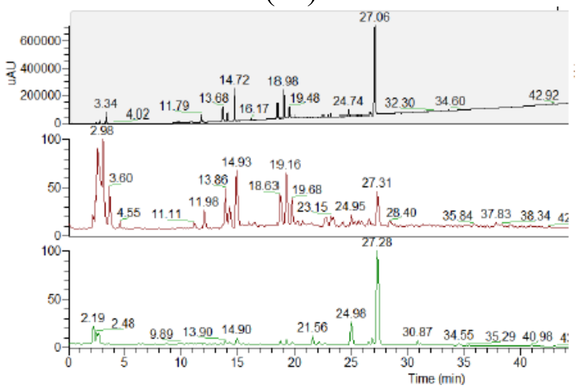

(25)

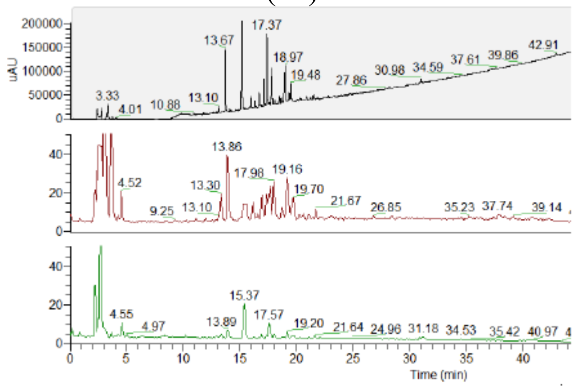

(26)

Figure S2. *Cont.*

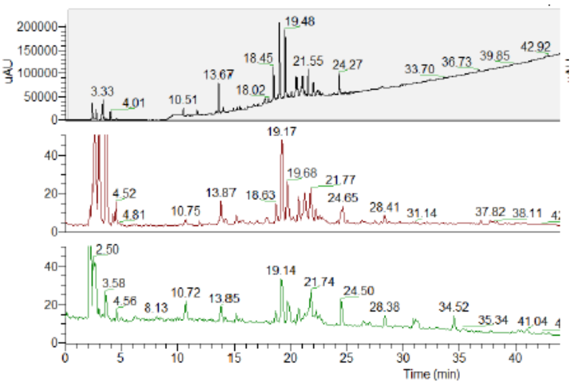

(**27**)

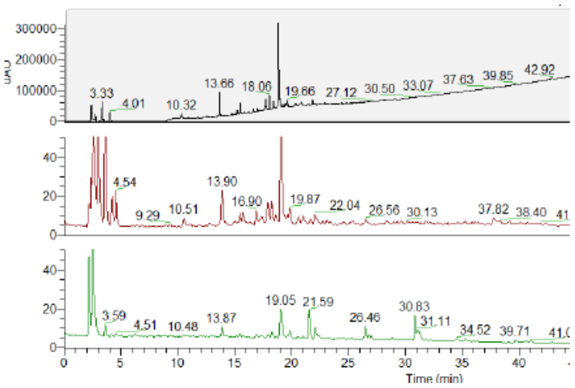

(**28**)

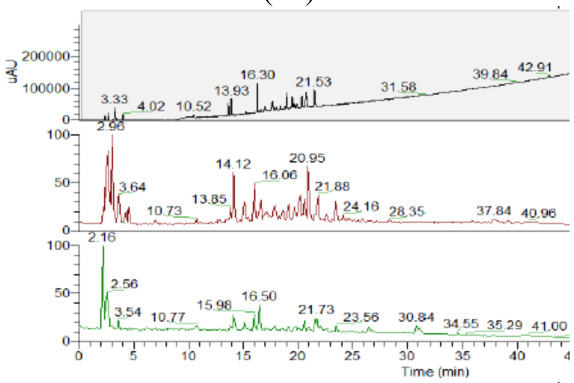

(**29**)

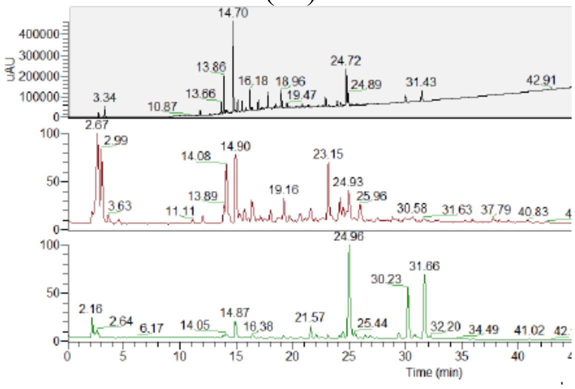

(**30**)

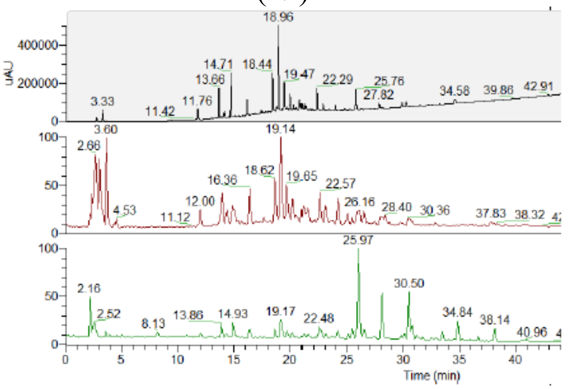

(**31**)

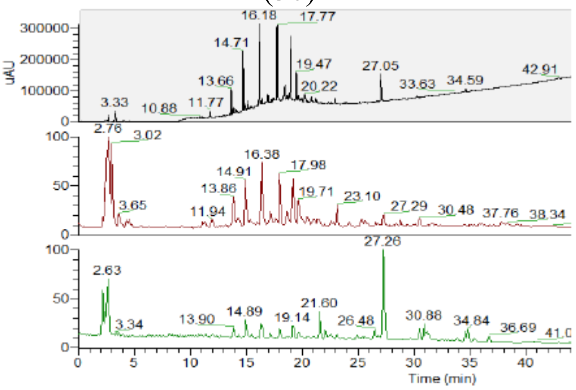

(**32**)

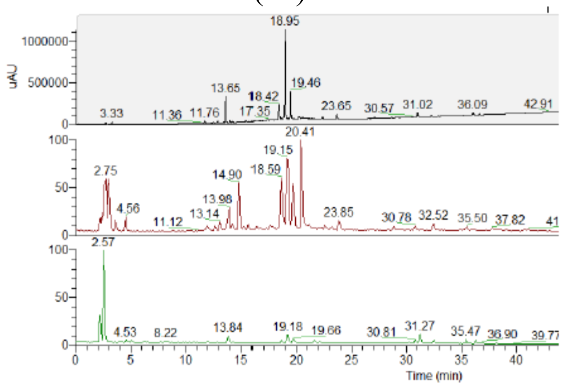

(**33**)

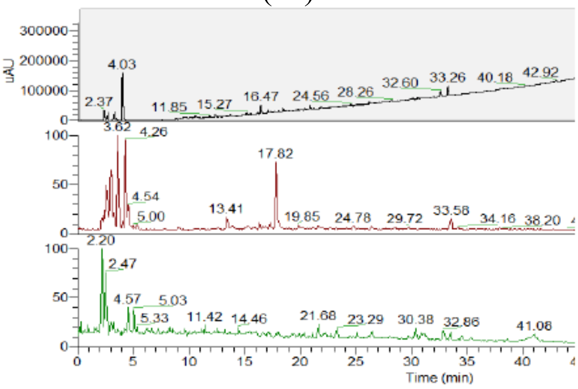

(**34**)

Figure S2. *Cont.*

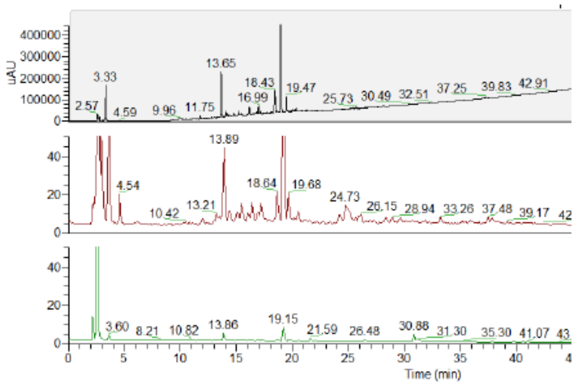

(35)

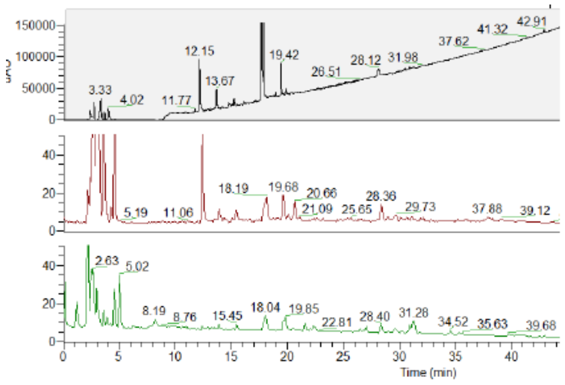

(36)

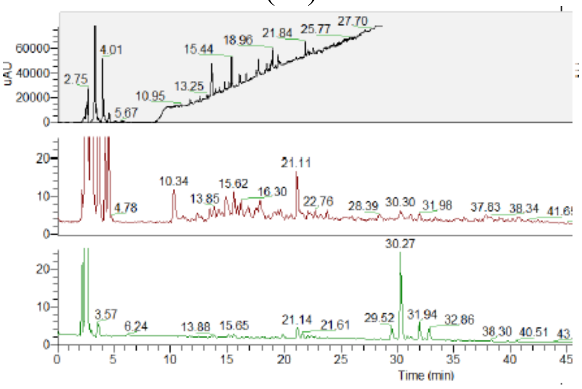

(37)

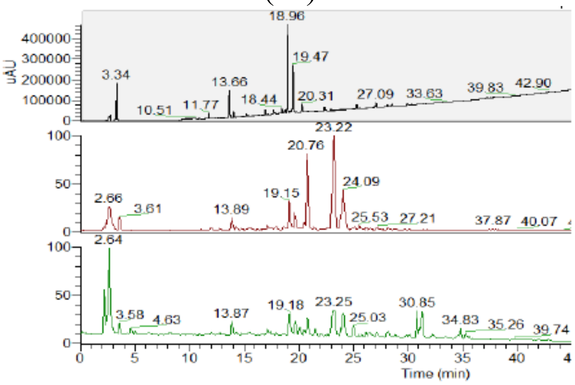

(38)

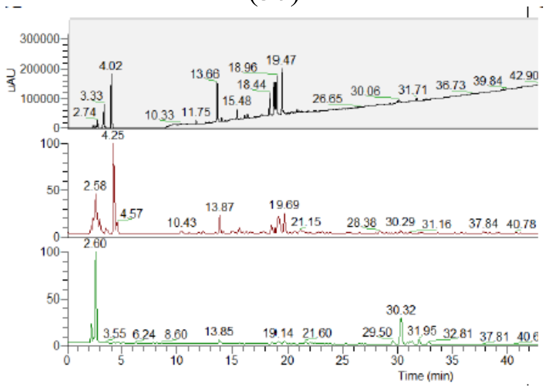

(39)

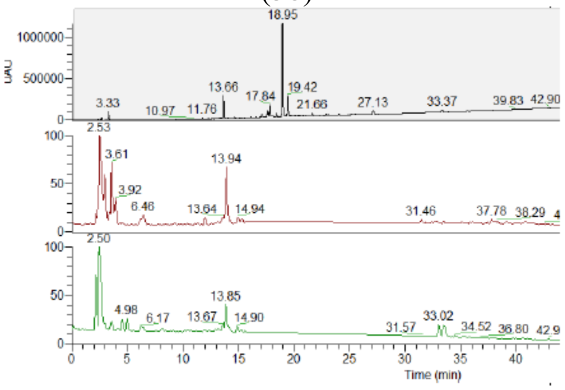

(40)

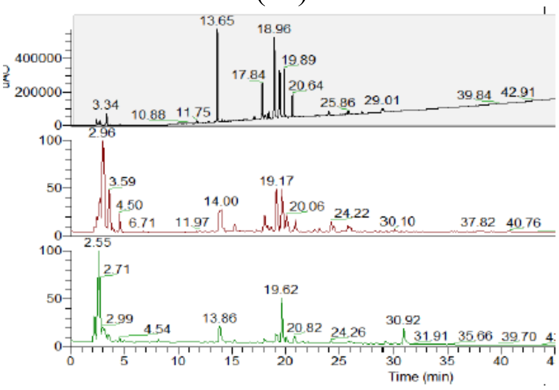

(41)

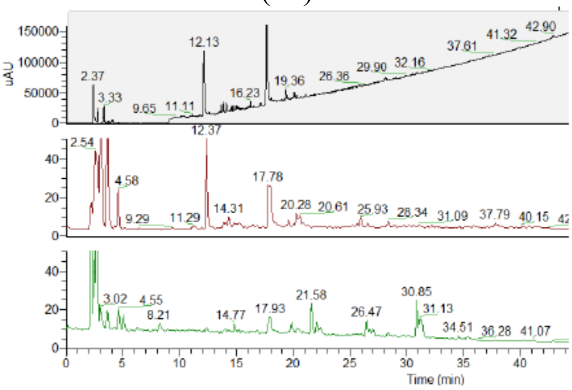

(42)

Figure S2. *Cont.*

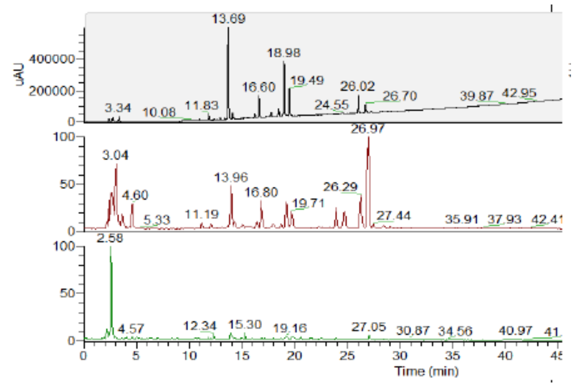

(**43**)

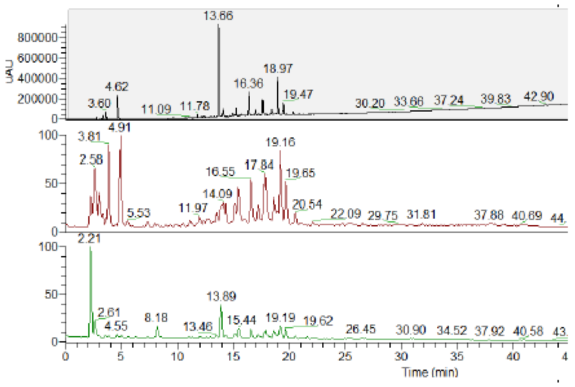

(**44**)

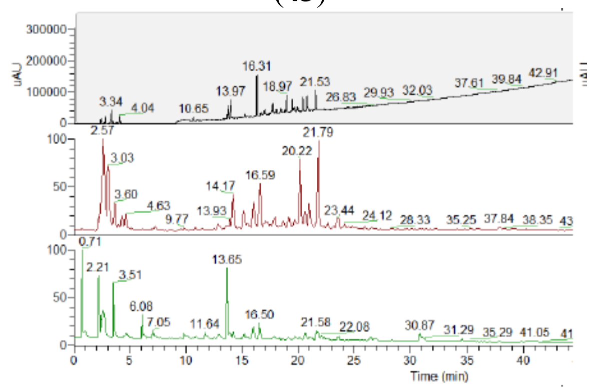

(**45**)

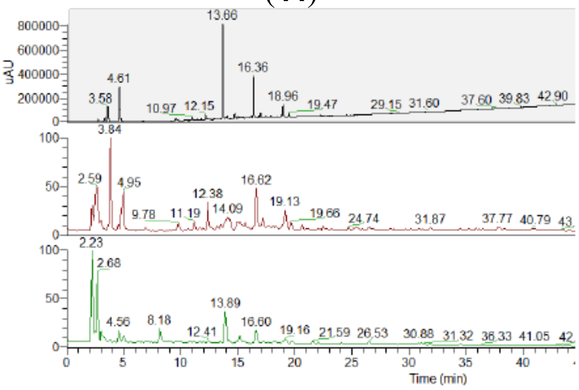

(**46**)

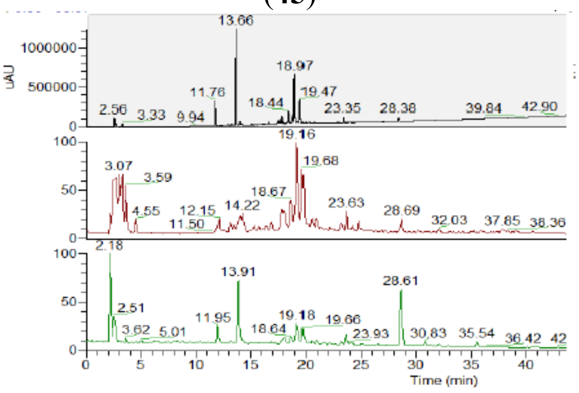

(**47**)

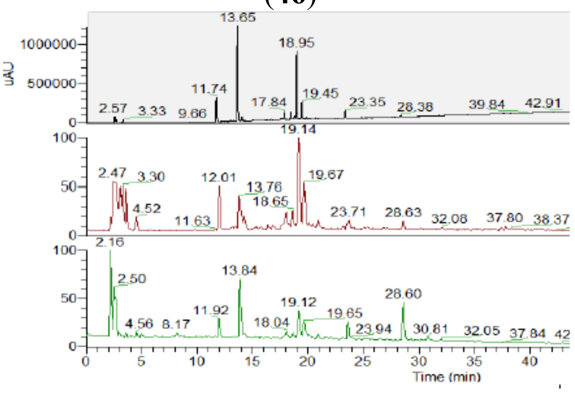

(**48**)

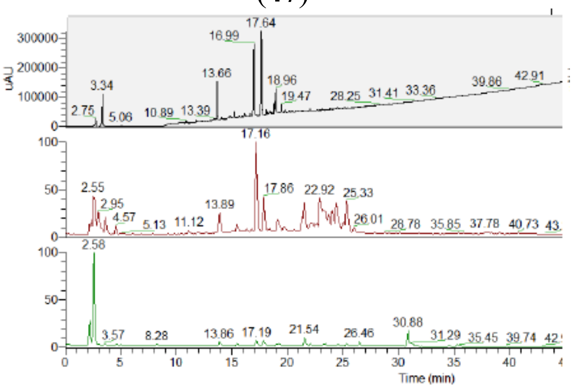

(**49**)

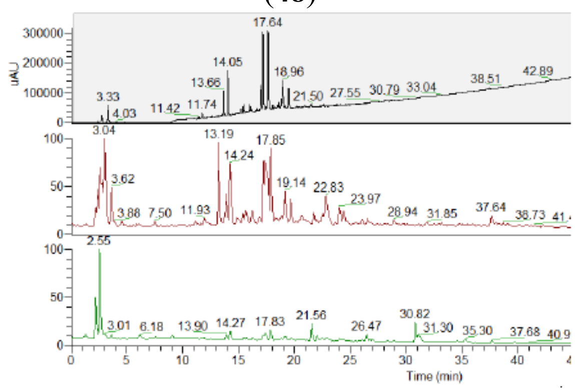

(**50**)

Figure S2. *Cont.*

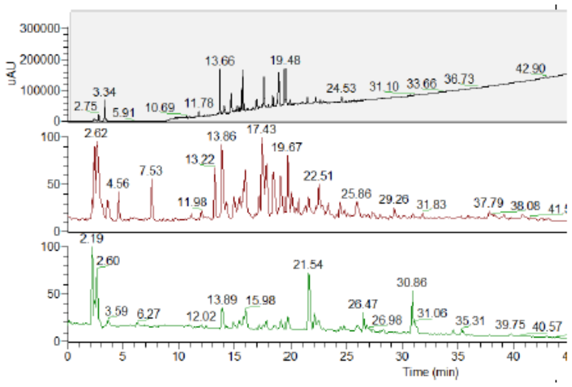

(51)

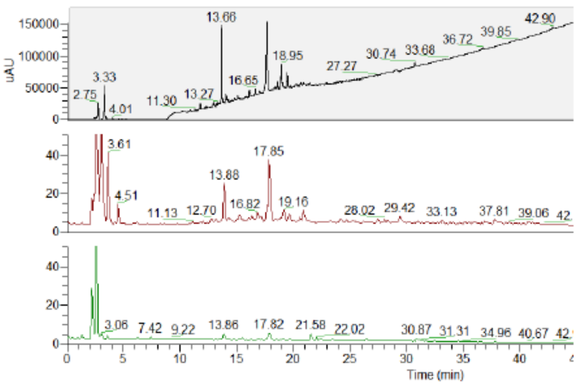

(52)

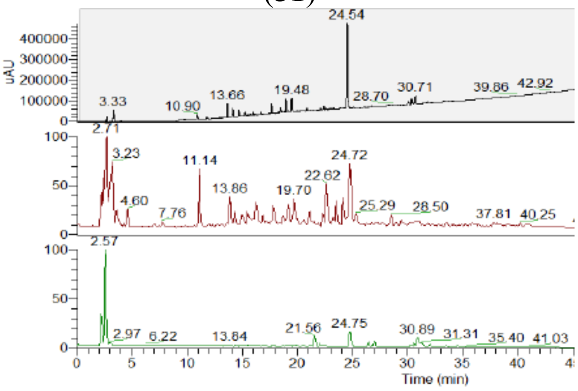

(53)

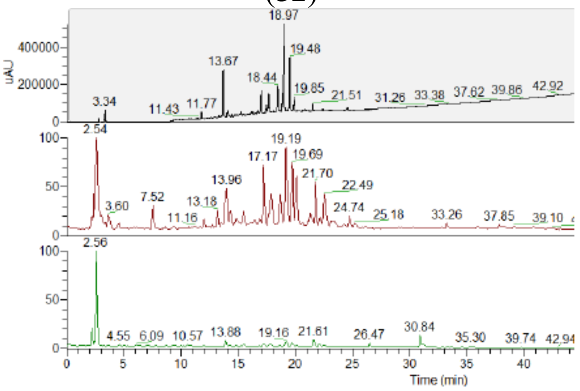

(54)

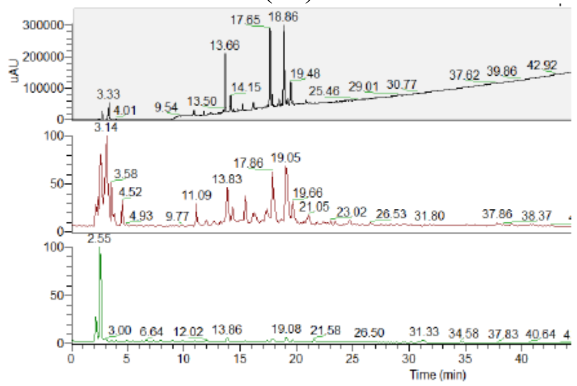

(55)

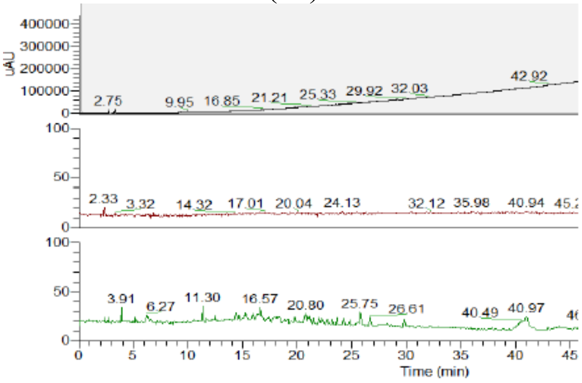

(blank)

Figure S2. Each sample has chromatograms detected using UV (wavelength at 254 nm) (top, black line), and full lock ms in negative (middle, red line) and positive mode (bottom, green line). Each sample has chromatograms detected using UV (wavelength at 254 nm) (top, black line), and full lock ms in negative (middle, red line) and positive mode (bottom, green line). The number of the samples are above each chromatogram.

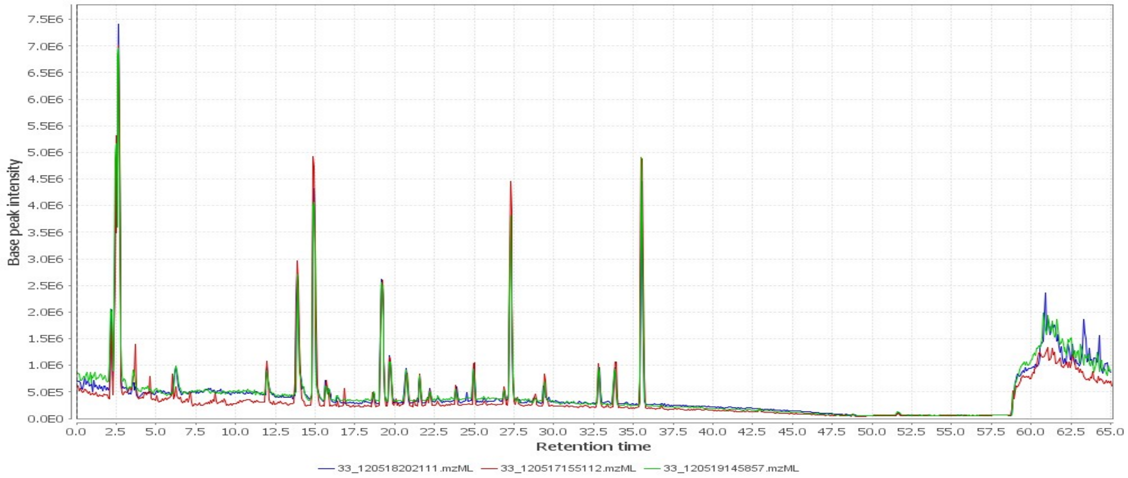

(**A**)

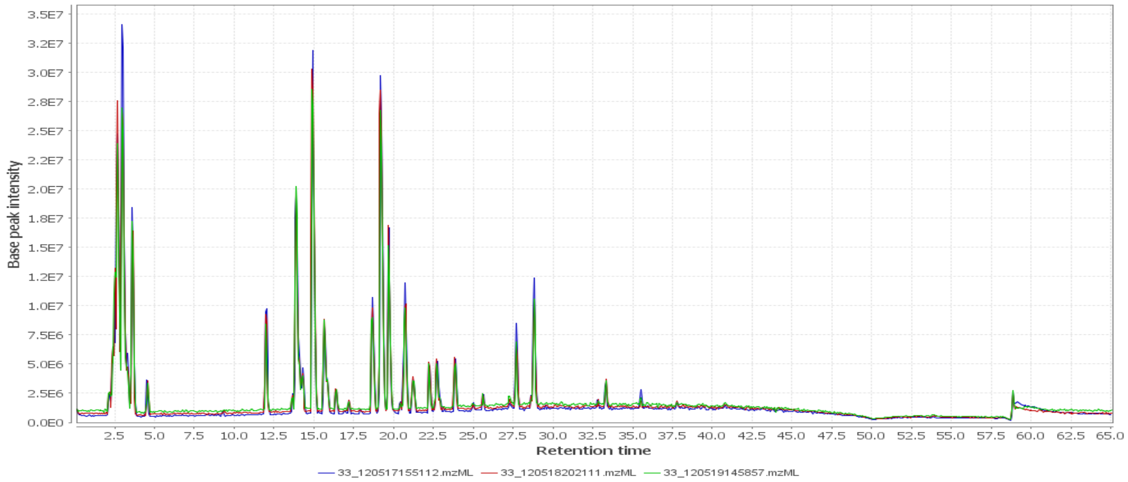

(**B**)

Figure S3. The same sample (#33) injected in the beginning, middle and end of the sequence of the injections, before data treatment. The overlap of the spectra shows the reproducibility of the analysis. The chromatograms were detected in the positive (**A**) and negative (**B**) mode.

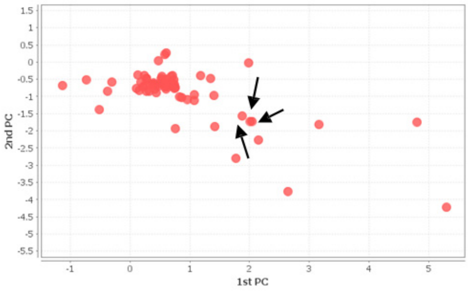

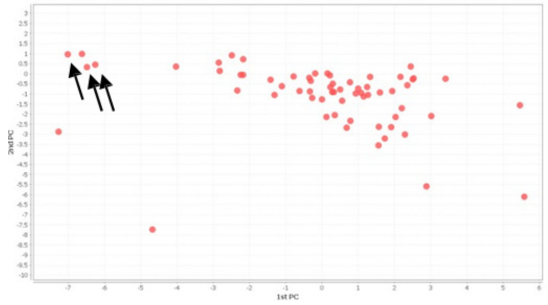

(**A**) (**B**)

Figure S4. The Principal Component Analysis—Expanded PCA plot of the positive (**A**) and negative; (**B**) mode MS datasets (before data treatment) for sample (#33) injected in the beginning, middle and end of the injection sequence indicates the reproducibility of the HPLC-ESI-HRMS analysis. The triplicate samples for extract #33 (indicated by the arrows) overlapped or grouped very near of each other.

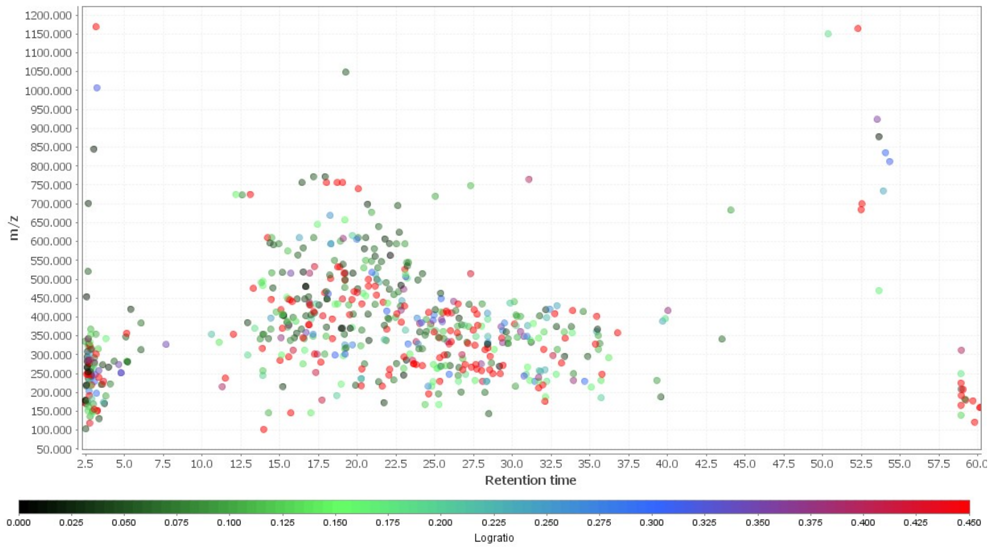

(A)

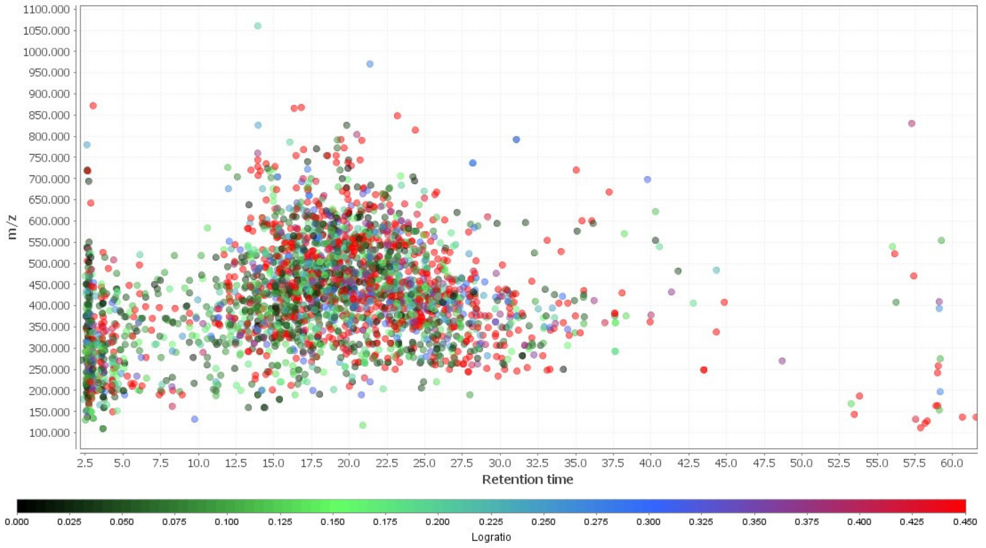

(B)

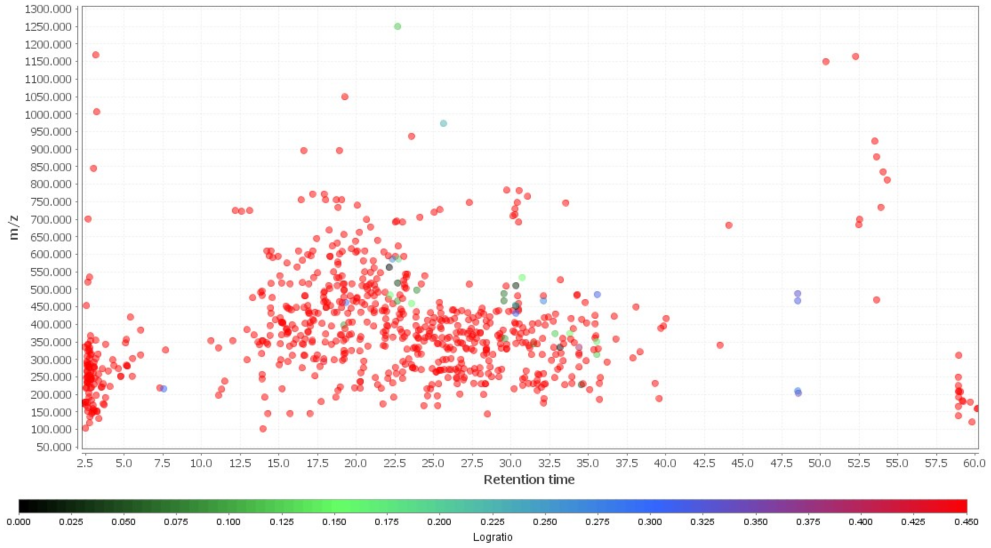

(C)

Figure S5. *Cont.*

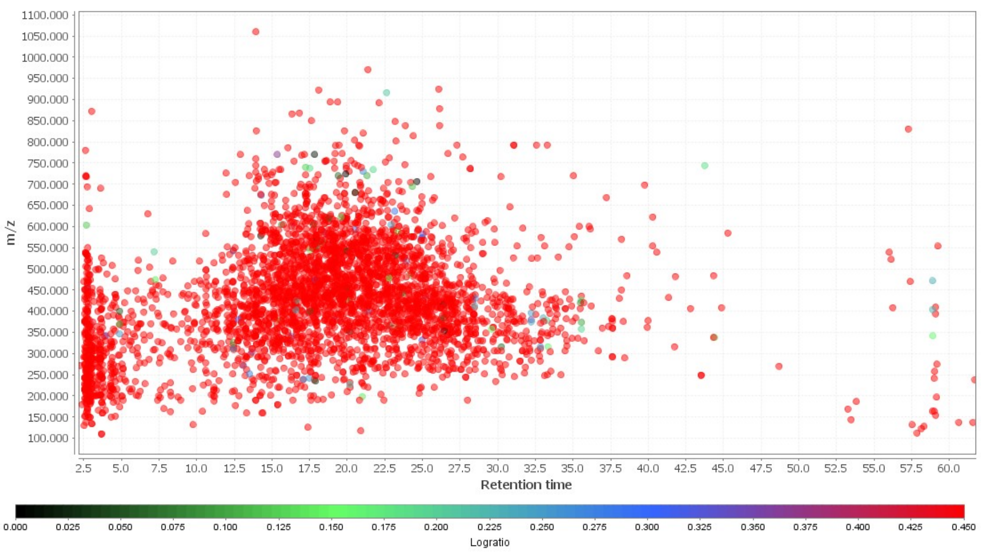

(**D**)

Figure S5. The correlation of variation analysis performed with the data from the three replicate injections of extract #33 (injected in the beginning, middle and end of the injection sequence of 57 extracts; before data treatment) validated the reproducibility of the results from the HPLC-ESI-HRMS analysis. Few attributes demonstrated variation as indicated by a log ratio > 0.40 (colored red on the heat bar), most of the peaks gained the green color (log ratio < 0.25) on the heat bar representing small variation as exhibited by the positive (**A**) and negative (**B**) mode datasets. The correlation of variation plots for the MS data with all the 57 samples (**C** for positive mode and **D** for negative mode) showed that almost all attributes afforded a log ratio > 0.4 and this result is coherent because variation between the different sample extracts must occur.

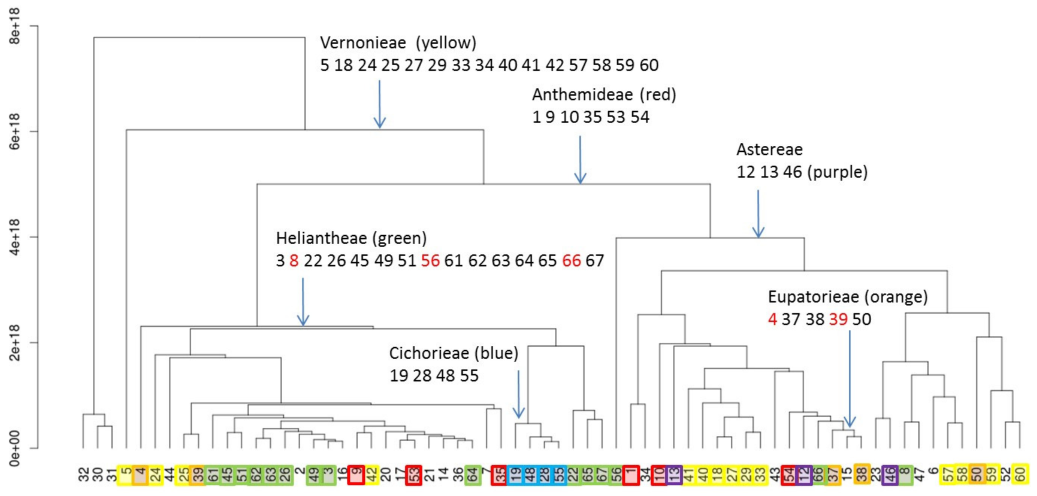

Figure S6. Hierarchical Cluster Analysis (HCA) of the metabolomics data of all Asteraceae species evaluated (n = 66, which included root, stem and flower parts for some species). Red highlighted sample codes represent extracts with unique chemistry when compared with other extracts within their respective taxa.

Table S1. A summary of available literature information on the chemistry of different plant species used in this study and AI activity [31] of their crude extracts. Those shown to exhibit dual inhibition of COX and LOX were highlighted in red.

Species	Sample Codes	Chemistry Investigated/AI Evidence	Tribes	5-LOX [31] (μg/mL)	COX-1 [31] (μg/mL)
Achillea millefolium Ledeb. [yarrow]	1	Yes/Yes	Anthemideae Cass.	(−)	(−)
Achyrocline satureioides (Lam.) DC. [macela]	2	Yes/Yes	Gnaphalieae (Cass.) Lecoq&Juill.	(−)	(−)
Acmella oleracea (L.) R.K. Jansen [toothache plant]	3	Yes/Yes	Heliantheae Cass.	(−)	(−)
Ageratum conyzoides L. [billygoat-weed]	4	No/Yes	Eupatorieae Cass.	(−)	(−)
Anteremanthus hatschbachii H. Rob.	5	No/No	Vernonieae Cass.	(−)	(−)
Arctium lappa L. [greater burdock]	6	Yes/Yes	Cynareae Less.	(+)	(−)
Arnica montana L. [arnica]	8	Yes /Yes	Heliantheae Cass.	(+)	(−)
Artemisia absinthium L. [wormwood]	9	Yes/Yes	Anthemideae Cass.	(+)	(−)
Artemisia annua L. [sweet wormwood]	10	Yes/Yes	Anthemideae Cass.	(−)	(−)
Baccharis dracunculifolia D.C. [alecrim do campo]	12	Yes/Yes	Astereae Cass.	(−)	(−)
Baccharis trimera (Less.) DC. [carqueja]	13	Yes/Yes	Astereae Cass.	(−)	(−)
Bidens pilosa L. [beggar-ticks]	14	Yes/Yes	Coreopsideae Lindl.	(−)	(−)
Calea cuneifolia DC.	15	No/No	Neurolaeneae Rydb.	(−)	(−)
Calendula officinalis L. [marigold]	16	Yes/Yes	Calenduleae Cass.	(−)	(+)
Chronopappus bifrons (DC. ex Pers.) DC.	18	No/No	Vernonieae Cass.	(−)	(+)
Cichorium intybus L. [chicory]	19	Yes/Yes	Cichorieae Lam. & DC.	(+)	(+)
Cynara scolymus L. [artichoke]	20	Yes/No	Cardueae Cass.	(−)	(+)
Dasyphyllum brasiliense var. latifolium (D.Don) Cabrera [espinho agulha]	21	No/Yes	BarnadesieaeD.Don	(−)	(+)
Echinacea purpurea (L.) Moench [purple cone flower]	22	Yes/Yes	Heliantheae Cass.	(+)	(−)
Emilia sonchifolia L. DC [lilac tassel flower]	23	No/No	Senecioneae Cass.	(−)	(−)
Eremanthus polycephalus (DC.) MacLeish	24	No/No	Vernonieae Cass.	(−)	(+)
Helianthus annuus L. [sunflower]	26	Yes/No	Heliantheae Cass.	(−)	(−)
Heterocoma gracilis Loeuille, J. N. Nakaj. & Semir	27	No/No	Vernonieae Cass.	(−)	(+)
Lactuca sativa L. [common lettuce]	28	Yes/No	Cichorieae Lam. & DC.	(+)	(−)
Lychnophora diamantinana Coile & S.B. Jones	29	No/No	Vernonieae Cass.	(+)	(−)
Lychnophora ericoides Mart. [arnica da serra]	33	Yes/Yes	Vernonieae Cass.	(+)	(−)
Lychnophora tomentosa (Mart. ex DC.) Sch. Bip.	34	No/No	Vernonieae Cass.	(+)	(−)
Matricaria chamomilla L. [chamomile]	35	Yes/Yes	Anthemideae Cass.	(+)	(−)
Mikania glomerata Sprengl. [guaco]	37	No/No	Eupatorieae Cass.	(+)	(−)
Mikania hirsutissima DC. [cipó cabeludo]	38	No/No	Eupatorieae Cass.	(+)	(−)
Mikania laevigata Schultz Bip. ex Baker [guaco]	39	No/No	Eupatorieae Cass.	(+)	(−)
Minasia scapigera H. Rob.	40	No/No	Vernonieae Cass.	(+)	(+)
Piptolepis monticola Loeuille	41	No/No	Vernonieae Cass.	(+)	(+)
Prestelia eriopus Sch. Bip.	42	No/No	Vernonieae Cass.	(+)	(+)
Pluchea quitoc D.C.	43	No/No	Inuleae Cass.	(−)	(−)
Smallanthus sonchifolius (Poepp. &Endl.) H. Robinson [yacon]	45	No/Yes	Heliantheae Cass.	(−)	(+)
Solidago microglossa DC. [arnica do campo]	46	No/Yes	Astereae Cass.	(+)	(+)

Table S1. *Cont.*

Species	Sample Codes	Chemistry Investigated/AI Evidence	Tribes	5-LOX [31] (µg/mL)	COX-1 [31] (µg/mL)
Sonchus oleraceus L. [sowthistle]	48	No/No	Cichorieae Lam. & DC.	(+)	(−)
Sphagneticola trilobata (L.) Pruskei	49	No/No	Heliantheae Cass.	(+)	(+)
Stevia rebaudiana (Bertoni) Bertoni [sweetleaf]	50	Yes/No	Eupatorieae Cass.	(+)	(−)
Tridax procumbens L. [tridax daisy]	51	No/No	Heliantheae Cass.	(+)	(−)
Tanacetum parthenium L. (feverfew)[34,36,37,69]	53	Yes/Yes	Anthemideae Cass.	(+)	(−)
Tanacetum vulgaris L. [tansy]	54	No/Yes	Anthemideae Cass.	(−)	(−)
Taraxacum officinale Weber ex FH Wigg. [dandelion]	55	Yes/Yes	Cichorieae Lam. &DC.	(−)	(+)
Tithonia diversifolia (Hemsl.) A. Gray [tree marigold]	56	No/Yes	Heliantheae Cass.	(+)	(+)
Vernonia condensata Baker [boldo baiano]	25	No/Yes	Vernonieae Cass.	(−)	(−)
Vernonia herbacea (Vell.) Rusby	57	No/No	Vernonieae Cass.	(+)	(+)
Vernonia platensis (Spreng.) Less.	58	Yes/No	Vernonieae Cass.	(+)	(+)
Vernonia polyanthes Less. [assa peixe]	59	Yes/No	Vernonieae Cass.	(+)	(+)
Vernonia rubriramea Mart. Ex DC.	60	No/No	Vernonieae Cass.	(+)	(+)
Viguiera arenaria Baker	61	No/No	Heliantheae Cass.	(+)	(−)
Viguiera bracteata Gardner	62	No/No	Heliantheae Cass.	(+)	(−)
Viguiera discolor Baker	63	No/No	Heliantheae Cass.	(+)	(−)
Viguiera filifolia Sch. Bip. Ex Baker	64	No/No	Heliantheae Cass.	(+)	(−)
Viguiera linearifolia Chodat & Hassl.	65	No/No	Heliantheae Cass.	(−)	(−)
Viguiera robusta Gardner	66	No/Yes	Heliantheae Cass.	(+)	(+)
Viguiera trichophylla Dusén	67	No/No	Heliantheae Cass.	(+)	(+)
**Reference inhibitors (RI)**					
Indomethacin (Sigma-Aldrich®)			RI of COX-1	(−)	(+)
Nordihydroguaiaretic acid (Sigma-Aldrich®)			RI of 5-LOX	(+)	(−)

Table S2. Peak area of the biomarkers of dual inhibition.

	Sample												
**ID**	**19**	**40**	**41**	**42**	**46**	**49**	**56**	**57**	**58**	**59**	**60**	**66**	**67**
Negative Mode													
671	0	1.78E+3	1.92E+6	3.70E+6	3.78E+2	1.11E+3	6.49E+3	2.01E+3	1.03E+4	3.16E+6	3.97E+6	1.51E+4	6.64E+2
694	0	5.14E+3	7.70E+3	4.80E+3	6.68E+1	1.14E+2	6.31E+2	1.41E+3	7.39E+2	5.12E+2	2.02E+2	3.91E+3	1.92E+2
2054	5.40E+2	4.57E+3	1.50E+3	5.92E+4	2.79E+3	1.87E+3	1.48E+7	1.86E+7	1.38E+4	5.46E+7	2.06E+7	2.81E+7	3.33E+6
2488	0	1.18E+3	1.69E+3	1.05E+3	0	5.70E+1	0	5.31E+1	1.17E+2	1.75E+2	6.47E+2	6.75E+1	2.22E+1
3913	1.02E+4	2.43E+3	1.04E+4	1.77E+4	1.03E+4	1.03E+4	2.48E+4	6.55E+6	6.43E+6	6.90E+6	3.60E+6	7.64E+6	3.64E+4
5001	2.63E+3	1.07E+3	1.50E+4	6.92E+3	2.00E+3	3.08E+4	2.70E+4	3.21E+4	7.06E+3	2.15E+3	8.24E+3	2.10E+4	1.25E+4
Positive Mode													
1190	0	9.06E+3	1.22E+2	2.39E+3	1.08E+1	0	0	3.18E+1	0	0	0	0	0
1273	0	2.27E+4	4.86E+4	1.61E+4	0	9.65E+1	7.17E+1	0	0	2.12E+1	1.40E+1	0	0
1282	0	1.54E+3	3.23E+3	2.39E+3	8.50	1.70E+1	9.36E+1	0	8.98	0	0	1.13E+3	0
1436	0	2.19E+2	4.45E+2	6.31E+3	0	0	0	0	0	0	0	0	0
1615	0	1.97E+3	4.31E+7	1.03E+5	6.07	1.88E+3	1.77E+3	9.46E+1	0	1.21E+2	4.15E+1	0	3.76E+1
1623	0	4.02E+2	1.25E+7	2.42E+3	0	2.20E+2	2.50E+1	0	0	1.04E+2	4.39E+1	0	0
1637	0	1.70E+3	6.59E+6	1.24E+3	0	3.74E+2	5.14E+6	0	0	0	0	9	0
